# Trends of litter decomposition and soil organic matter stocks across forested swamp environments of the southeastern US

**DOI:** 10.1371/journal.pone.0226998

**Published:** 2020-01-03

**Authors:** Beth A. Middleton

**Affiliations:** U.S. Geological Survey, Wetland and Aquatic Research Center, Lafayette, LA, United States of America; Sichuan Agricultural University, CHINA

## Abstract

A common idea in the discussion of soil carbon processes is that litter decomposition rates and soil carbon stocks are inversely related. To test this overall hypothesis, simultaneous studies were conducted of the relationship of environmental gradients to leaf and wood decomposition, buried cloth decomposition and percent soil organic matter in *Taxodium distichum* swamps across the Mississippi River Alluvial Valley (MRAV) and northern Gulf of Mexico (GOM) of the US. Decomposition of leaf tissue was 6.2 to 10.9 times faster than wood tissue. Both precipitation and flooding gradients were negatively related to leaf and wood litter decomposition rates based on models developed using Stepwise General Model Selection (MRAV vs. GOM, respectively). Cotton cloth should not be used as a proxy for plant litter without prior testing because cloth responded differently than plant litter to regional environmental gradients in *T*. *distichum* swamps. The overall hypothesis was supported in the MRAV because environments with higher precipitation (climate normal) had lower rates of decomposition and higher percent soil organic matter. In the MRAV, higher levels of percent soil organic matter were related to increased 30-year climate normals (30 year averages of precipitation and air temperature comprising southward increasing PrinComp1). Soil organic carbon % in inland vs. coastal *T*. *distichum* forests of the MRAV were comparable (range = 1.5% to 26.9% vs. 9.8 to 31.5%, respectively). GOM swamps had lower rates of litter decomposition in more flooded environments. Woody *T*. *distichum* detritus had a half-life of up to 300 years in the MRAV, which points to its likely role in the maintenance of inland “teal” soil organic carbon. This unique study can contribute to the discussion of approaches to maintain environments conducive to soil carbon stock maximization.

## Introduction

A better understanding of the relationship of environment to soil carbon processes across geographical gradients is helpful to support models of the effects of climate and land-use change on ecosystems, especially because more carbon is stored as soil organic matter than in the atmospheric pool [1–2]. Because wetlands store more soil carbon than their terrestrial counterparts, wetlands may take center stage in future discussions of climate mitigation policy and national greenhouse gas inventories [3–4], yet wetlands remain understudied. While general soil carbon maps have been developed for the world [5], these maps are lacking detail to show the nuances of wetland responses to environment [6–7]. One exception is for saline wetlands, where soil carbon density levels decrease with increased mean annual air temperature southward [8].

Because wetlands are highly influenced by their hydrology as related to precipitation and other factors, carbon processing and storage in wetlands differs in critical ways from better known terrestrial ecosystems [9]. In flooded wetlands, decomposition may be slow and carbon accumulation high because of low oxygen conditions [10] while well aerated but moist environments can have the fastest decomposition [9]. In flooded conditions where production levels remain high, organic matter can accumulate in soil rapidly [1], especially in floodplain depressions, which remain flooded for longer periods of time [11]. Also, coastal wetlands with higher salinity often have lower decomposition rates than inland wetlands with lower salinity [12–14] but not for all tissue types such as roots [15].

While environment is undoubtedly an important driver of decomposition rates, tissue litter quality may also be important [16]. Woody stem and root material decompose more slowly than leaves because of relative tissue toughness i.e. force needed to break the material [17] and presence of lignin [18]. Relevant in this study is that *Taxodium distichum* tissue is high in tannin and may decompose slowly [19–20]. Tissue decomposition may slow over time if it enters a recalcitrant phase after a period of years [21]. Such differences in tissue decomposition rates can be quite important in carbon accumulation pattern. For example, mangrove islands comprised of peat atop old coral reefs in Belize are comprised mostly of dead roots, which decompose slowly [16]. Therefore, environmental shifts in tissue composition could have major consequences for carbon cycling and accumulation [18]. To minimize differences in decomposition rates between species and/or tissue types, some researchers use standardized materials such as cellulose or cloth strips [22].

Beyond decomposition rates, primary production may have a major influence on soil carbon stocks [9], noting that primary production levels often shift across the geographical and climate temperature range of species [23]. Geographical patterns in plant production are relatively well described; herbaceous wetlands often decrease with temperature in production level linearly from equator poleward [24–26]. In contrast, the geographical production trends in *T*. *distichum* swamps in the southeastern United States are highest at mid-latitude (e.g., in Arkansas) with lower levels to the north and south [23], with potential implications for soil carbon storage. Percent soil organic matter also is influenced by certain factors such as mineral input, especially in floodplain wetlands [27]. Also, latitudinal patterns may be more nuanced in wetland than in their terrestrial counterparts, perhaps because of changing local environments (e.g., hydroperiod [6] or coastal salinity [8, 28].

Despite the fact that factors other than decomposition rate may determine geographical differences in soil carbon stocks, many studies assume that there is a direct connection between soil carbon stock and decomposition rate [8, 10]. The objective of this study was to compare linked field responses of litter decomposition, cloth decomposition and percent soil organic matter in similar temporal, geographical (latitude, longitude, site type; see S1 Table for variable details) and environmental settings within *T*. *distichum* swamps along the Mississippi River Alluvial Valley (MRAV; inland type), and/or northern Gulf of Mexico (GOM; tidal and nontidal coastal types). This study investigated if these patterns might be influenced by climatic (air temperature and precipitation) and/or site water variables (depth, flood/drawdown duration and salinity). The following hypotheses were tested.

**Fig 1:**
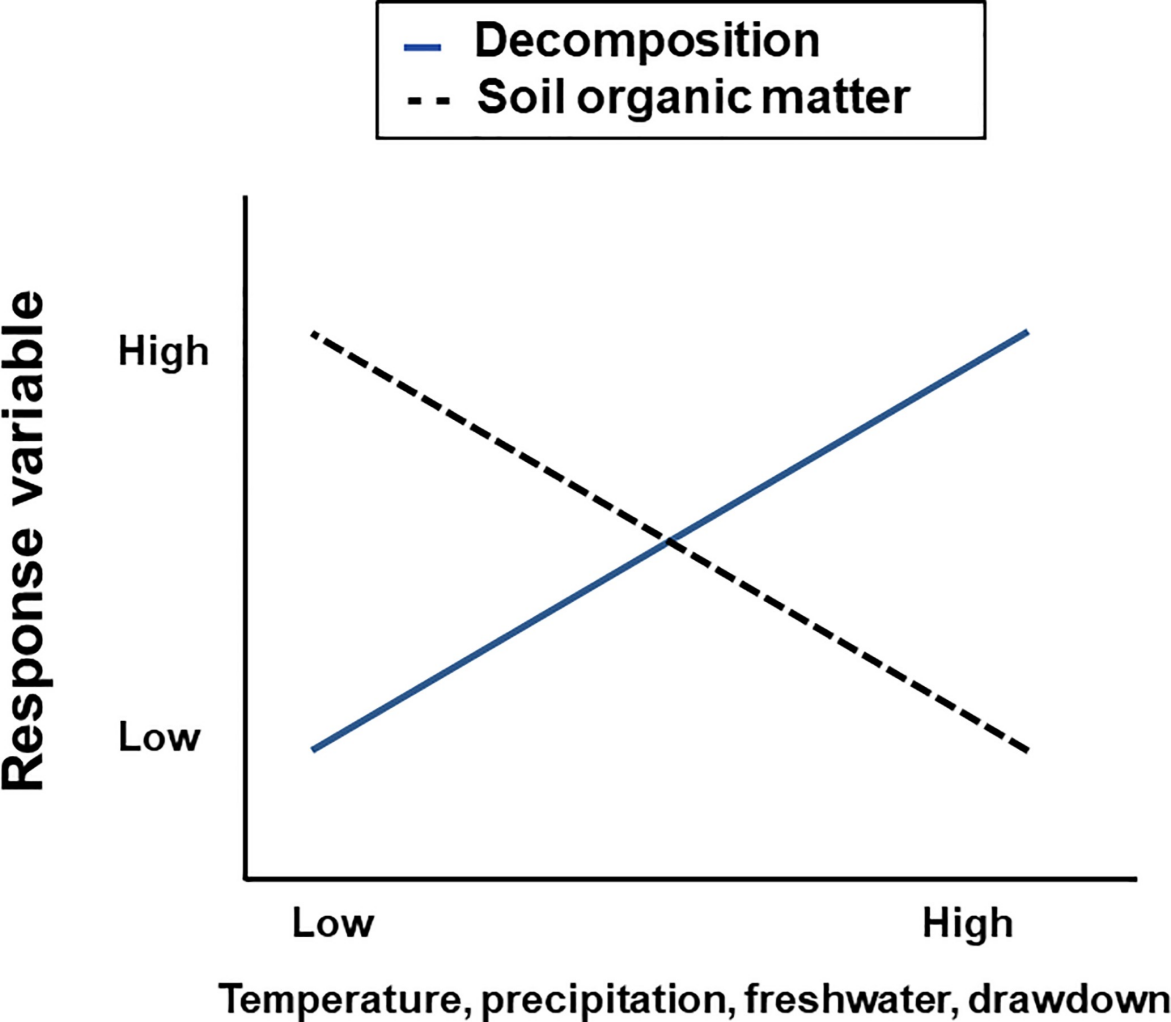
Hypothesized relationships of plant litter and cloth decomposition rates (solid blue line), as a logical opposite process to the accumulation of percent soil organic matter (dashed black line) across climatic and environmental gradient of temperature, precipitation, freshwater (vs. salinity) and drawdown (mathematical complement of flooding). For the purposes of this hypothesis, freshwater often is linked to higher levels of litter decomposition, and is an environment that could lower soil organic matter accumulation. While no literature could be found supporting freshwater in this role, the idea is presented here as part of the related hypothesis.

*Taxodium distichum* tissues (leaf and wood) and cotton cloth decomposition will have a positive relationship to climatic factors such as air temperature and precipitation [1, 29], and local environments of drawdown (mathematical complement of flooding), and freshwater level (i.e., low salinity; Fig 1A) [10]. Percent soil organic matter will have negative relationships with climatic variables such as air temperature [8], precipitation [in conjunction with increasing temperature) [6], drawdown (mathematical complement of flooding) [10], and salinity [13–14] (Fig 1).

## Materials and methods

Permits for all field research have been acquired through the US Fish and Wildlife Service, US National Park Service and other government agencies. These are in the form of letters to the author and can be provided by request.

### Study area and design of the North American Baldcypress Swamp Network

**Fig 2:**
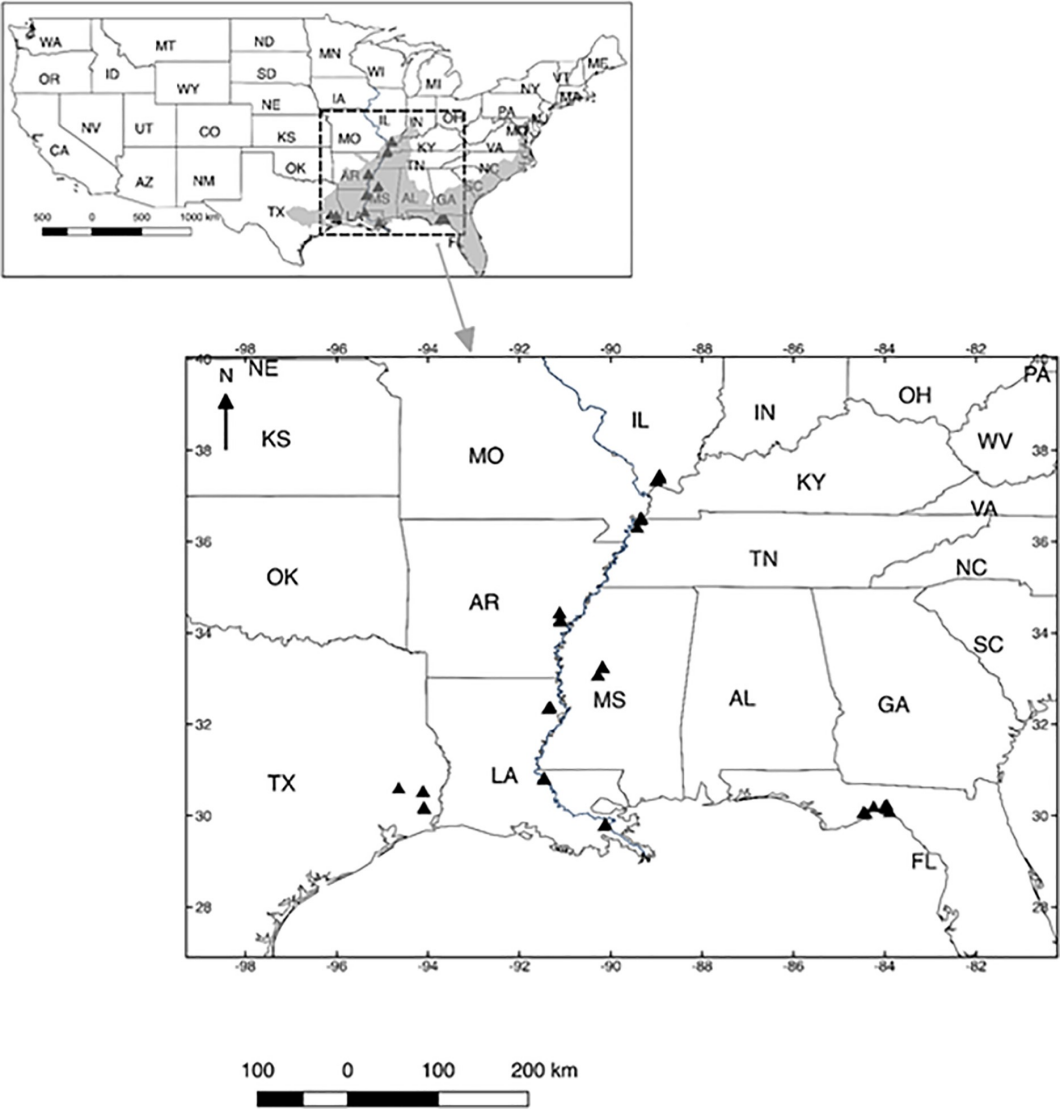
North American Baldcypress Swamp Network location in the southeastern United States (gray shading depicts the range of *T*. *distichum* var. *distichum* [71]. These freshwater forested swamps lie along the Mississippi River Alluvial Valley (MRAV; inland swamps) and Gulf of Mexico Coast (tidal and nontidal), and are dominated by *T*. *distichum*. Each network site has five replicate swamps (triangles), with three-five sites selected for the leaf and wood litter decomposition and cloth-strip decomposition studies. Two sites were selected in swamps along the MRAV to examine percent soil organic matter. See S2 Table for details on network location and specific sites.

Across the *T*. *distichum* swamp region of the southeastern United States, decomposition rates and organic matter deposition were studied in nine geographical areas (3–5 swamps each; see S2 Table) in the North American Baldcypress Swamp Network. Inland swamps of the MRAV were located from southern Illinois to central Louisiana (6 MRAV sub-states: IL, TN/KY, AR, MS, NLA, CLA; 37.43° to 29.79° N; Fig 2; see S2 Table for site and collection details). Coastal swamps were included tidal and non-tidal types along the GOM from eastern Texas to the Panhandle of Florida (3 GOM sub-states: TX, SLA/CLA, FL; 94.64° to 83.89° W; Fig 2, S2 Table). A total of 43 sites were used in the study (S2 Table). Locations referred to as mid-range in this study include those nearest White River NWR in Arkansas (~34.2 to 34.4 ^o^N latitude). During the study, mean annual air temperature (daily mean temperature; S1 Table) in these MRAV and GOM swamps (2007 vs 2011, respectively) ranged from 13.7–19.0°C and 19.7–20.2°C, respectively. Total annual precipitation ranged from 1279–1655 mm y^-1^ and 1551–1655 mm y^-1^, respectively [30]. Coastal swamps of the GOM lie along an increasing gradient of normal total annual precipitation (average over 30 years of total annual precipitation; normal annual precipitation; S1 Table and S2 Table) from west to east [30], although those patterns are not annually consistent [31]. Clay and silt mixtures predominated the soil types of these swamps (S3 Table) [32–33].

Inland swamps in the network are seasonally flooded during the winter from approximately December through May, with much interannual variation in annual % time of drawdown (S1 Table and S2 Table) [34]. Both Cat Island NWR and White River NWR are on floodplains adjacent to major rivers and often experience winter flooding of 6–10 m. Coastal tidal swamps are micro-tidal and typically flooded, especially in southerly winds [34]. Both coastal and inland swamps are on un-impounded floodplains, and relatively unmodified from a hydrologic perspective with flood pulsing from the river. Exceptions are swamps at NLA in the Tendal River in Tensas National Wildlife Refuge (NWF), where the channel is cutting downward through the bed of the channel (i.e., downcut) with only occasional bank overflow (Middleton personal observation). Each location in the network has a nearby USGS/USACOE or state-operated water gage (S2 Table).

These mature secondary growth forests of the network had fully developed canopies in a strand at least four trees wide, although swamp widths generally are narrower in the southwestern part of the range (i.e., southeastern Texas). Sporadic individuals of *T*. *distichum* along rivers at southwestern extreme of the range were excluded because these narrow strands did not fit the criteria for this study. The dominant woody species of these swamps were *T*. *distichum* var. *distichum* with *Cephalanthus occidentalis* (buttonbush), *Fraxinus pennyslvanica* (green ash), *Myrica cerifera* (southern waxmyrtle), *Nyssa aquatica* (water tupelo), and *Quercus lyrata* (overcup oak) [34–35]. Regarding disturbance patterns in these forests, none of these forests had been logged for more than 60 years. All of the sites show signs of burning at some time in the past, and remnants of cut cypress stumps (Middleton, personal observation). The forests in both southern Illinois and Tennessee experienced branch damage during ice storms (Middleton, personal observation in 2013), and the coastal forests are subject to occasional wind damage or salinity intrusion from hurricanes [34]. The study sites were on public property managed mostly by the Department of Interior (National Park Service (NPS), Fish and Wildlife Service (FWS)), and a few swamps on state lands in Illinois and Florida (S2 Table). The selected sites represent some of the most pristine *T*. *distichum* forests in the southeastern United States.

### Study site design and sampling

At each geographic location, 3–5 sites were selected for the decomposition studies and 2 sites for the soil coring of percent soil organic matter (S2 Table). At each site/swamp, five replicate plots were established along a 125 m transect in stratified random positions within 25 m intervals, and each plot marked with a wooden stake [35]. Regarding the nature of experimental units in the study and any pseudo-replication of samples, note that the five plots for decomposition and soil cores were selected randomly at these stratified random points. These five plots at each site were independent samples and not pseudo-replicates because these were far enough apart to not influence the other samples.

### Leaf and wood litter decomposition

Leaf and wood decomposition rates were tested separately using both one-year and two-year litter bag study. Litter bags (10 x 10 cm) were constructed of fiberglass mesh (1 mm^2^; Brinson et al. 1981) and each bag was filled separately with 5 g of newly senesced, air-dried leaf or wood material (weighed to within 0.00 g; oven dried (70 ^o^C) leaves and wood weighed 4.06 vs. 4.30 g, respectively). Leaf material was collected from *T*. *distichum* trees growing on the property of the Wetland and Aquatic Research Center in Lafayette, LA, which reduced any confounding effects of regional intraspecific variation in litter quality. Wood material was commercial cypress wood mulch. While mulch is a widely available, uniform and easy to allocate to litter bags, it should be noted that small pieces of cypress wood are likely to decompose more quickly than larger logs. Five litter bags of each tissue type were placed on the surface of the ground tied with a fishing line to separate stakes at each of five plots per site. In flooded situations, the deployed litter bags sank to the surface of the soil within one day. At each site, litterbags were placed in the field on the same day, but the precise timing of deployment and collection across locations were staggered slightly for logistical reasons (S2 Table). In all studies, one bag was collected from each plot (five plots transect^-1^) at various time intervals after deployment including approximately 0, 90, 180, and 365 days in the MRAV and GOM (2007 vs. 2011, respectively; S2 Table). Litter bags were also collected after ~ 730 days, noting that the litterbags increasingly disappeared from plots over time because of floods, tidal surge, tree fall and animal damage (e.g., bear and raccoon). At the time of collection, a single litter bag was removed from the plot stake, placed into a marked resealable bag, and transported to the lab in a portable cooler. Litter remaining in the bag was washed and rinsed in distilled water, oven dried at 70 ^o^C to a constant weight. Litter was weighed to within 0.00 g (beginning) and 0.0000 g (ending) weight.

The leaf and wood litter decomposition study started in September 2007 in MRAV locations (inland; 3 sites per location) and in April 2011 in GOM locations (coastal tidal and nontidal; 3–5 sites per location). As part of the GOM study in 2011, the CLA (Cat Island, CINWR) and SLA (Jean Lafitte National Historic Park and Preserve, JNLHP&P) locations were sampled. See S1 Table for abbreviations and definitions of response, climate, environment and geographic variables for these studies.

### Cloth decomposition

Relative rates of decomposition were determined using a cotton strip assay method [36]. The approximately 10-day long, cloth decomposition study was installed in the same sites and transect plots as the leaf and wood litter decay study starting in September 2007 in the MRAV (inland) and in April 2011 in the GOM locations (coastal tidal and nontidal)(S2 Table). Cloth strips were deployed within 0.5 m of each plot marker along NABSCN transects. Strips of artist cloth (10 x 35 cm; Fredrix 12-ounce Duck Artist Canvas, Style 548) [37] were slid vertically into a slightly more than 30 cm deep hole cut with a sharpshooter shovel (flattened shovel; 35.6 cm long x 12.1 cm wide x 1 cm thick blade) within 0.5 cm of the plot marker, and then an adjacent slit made and the sides pressed closed. Each deployed strip was marked with a piece of flagging. The strip was marked at the ground level with a permanent marker, with 5 cm of the strip projecting from the hole. Five control strips were immediately removed from the holes, and transported to the lab in a portable cooler for analysis. Test strips were retrieved from the holes after approximately 10 days, and subsequently washed in deionized water, wrapped in foil, and transported to the lab in a portable cooler. Strips were dried on a clothesline and stored dry in a resealable bag. To test tensile strength, 15–2 x 10 cm sub-strips were cut from top to bottom of the strip. After rewetting the sub-strips in deionized water for two or more minutes, cloth tensile strength at each depth was measured in Newtons (N) with an industrial fabric tensometer (Mecmesin basic force gauge BFN1000N), which was mounted on a MultiTest1-*d* motorized test stand.

### Percent soil organic matter

Using a Russian peat corer, soil was cored to a depth of 100 cm within 5 m of each plot at two sites in each geographic location along the MRAV only (i.e., five cores per site) from September 6–16, 2007 (S2 Table). Location CLA (Cat Island NWR) was omitted because the soil was too compacted to core. The extracted core from each plot was carefully divided into 10 cm sections in the field, each section placed into a separate resealable bag, and transported in a portable cooler. To determine percent soil organic matter, three sub-samples from each soil section were weighed (wet weight) separately, dried at 105°C for 4 hours, weighed again, ashed in a muffle furnace at 450°C for 8 hours, and weighed a final time [38].

### Climate and environment of location and site

Climate data for the period of the field studies were downloaded from the weather station [31] nearest to each location, using ~365 days for the leaf and wood decomposition study based on the number of days the litter was in the field during the first year study, and 8–10 day for the cloth decomposition study based on the number of days the cloth was in the field (S2 Table). Distance from the site to the nearest weather station was determined using [39]. Daily, annual and normal (1981–2019) (NOAA 2018 climatic variables (short name in brackets; S1 Table) included: mean daily annual precipitation (mm) [daily precipitation], mean daily maximum air temperature (^o^C) [daily maximum temperature], mean daily minimum air temperature [daily minimum temperature], mean annual air temperature [annual air temperature], normal total annual precipitation [normal annual precipitation] (mm), normal mean annual air temperature [normal annual temperature] (S1 Table). Means of climate data were constructed based on the number of days with data for the time period of interest [40]; any flagged data were removed from the data set. The same climate data was used for the MRAV litter decomposition and percent soil organic matter analysis compared on an annual basis. Also, climate normals for the period 1981–2010 were constructed for an additional analysis of percent soil organic matter [41]. Daily and annual climatic variables were used in analysis of the litter decomposition and cloth decomposition data. Annual and normal climatic variables were used for analysis of the percent soil organic matter data. All climatic variables were calculated for each location [40]. Environmental variables measured at each plot included: pore water salinity (ppt; salinity), day-of-visit water depth (cm; water depth), and annual percent time of drawdown (drawdown % or time not flooded in decimal percent; S1 Table and S2 Table). Pore water salinity was measured using a pore water sipper and collected water was frozen in a portable freezer, and salinity later measured with a YSI EC 300® probe. Water depth was measured with a meter stick in each plot in each site on each day of visit (S2 Table).

Drawdown % was calculated separately for each plot at each site by using day-of-visit water depth to estimate daily changes in water elevation by matching plot depths to the nearest continuously recording hydrologic gauge [34–35, 42–43] (S2 Table). Water depths were recorded at monuments associated with Sediment Elevation Tables (stationary elevation markers) [44]) and matched with plot water depths during the site visit with the “flood date” designated as the deepest recorded “flood” during field work from 2002–2019 (S2 Table). These monuments are helpful to determine any shift in plot elevations over time due to a tree fall, erosion or sedimentation event because permanent benchmarks do not readily change in elevation. To determine the mean percent of time a plot was drawn down during the study period (~ 1 year), daily mean water depth and elevation at each gauge were compared to the period estimated for the plots. The plot was designated as ‘‘drawn down” on a given day depending on whether the water depth was less or more than one cm, respectively, a condition that might affect the aeration level of the plot. Overall percent time of drawdown was set as the percent of days during the study that the plot was drawn down vs. the total number of days with gauge records. Means for the climate and drawdown data were calculated during for the first year of the litter decomposition study by determining the daily status of plots during the study (drawdown %) for each site [40] (S2 Table). There was no attempt to determine the percent amount of soil water that originated from atmospheric precipitation versus other sources (e.g., groundwater).

### Statistical analyses

Leaf and wood litter decomposition. Leaf and wood decomposition coefficients (k) were calculated using a single exponential model, using the equation for decomposing material as: y_t_ = yoe-kt, where y_t_ = the biomass at time t, yo = the initial biomass, and t = time [45], with the data fitted to a negative exponential curve [46]. To calculate k, ln[decimal % remaining] is linearly regressed with time in decimal parts of a year [47], or one year in the case of this study. The rate of decomposition, k, equals the slope of this relationship with all data points fitted to the regression line [46]. The equation for the half-life of the litter is: 0.693/k [46]. In this context, the half-life (50% turnover time) is the time necessary for one half of the material to decompose and is conceptually similar to an LD50. Note that covariance estimates show that the litter decomposition rates were not correlated at plots within a site (p = 0.12309 [48], so that the litter decomposition study was not pseudo-replicated.

#### Cloth decomposition

Cotton tensile strength loss (CTSL) of the field decomposed cotton strips was compared to control sub-strips (field inserted and removed) as: CTSL (%) = [1 –(N/C)] x 100, where N was the tensile strength of the decomposed cloth and C was the mean of the tensile strength of the control sub-strips cloth in Newtons. The rate of cloth decomposition was calculated as the percent loss in tensile strength per day [36]. CTSL values were log transformed to meet the assumptions of normality and homogeneity [49]. CTSL responses from 0–30 cm soil depths were combined into means of upper and lower layers as based on a two-way ANOVA performed on log transformed data compared at 10 cm intervals (two groups: 0–10 vs. 10–30 cm depth; one-degree-of-freedom contrasts: p < 0.05) [40]. Note that covariance estimates show that the upper and lower layers were not correlated at a site (p = 0.9578) [48], so that cloth decomposition samples within a site and between depths were not pseudo-replicated under the normality assumption.

#### Percent soil organic matter

Mean of the three sub-samples of percent soil organic matter from each 10 cm core section were evaluated statistically. Other conversions were made to percent soil organic carbon by multiplying percent soil organic matter by 0.45 [50] to facilitate comparison to other studies. Bulk density was determined as: oven dry sample weight divided by sample volume. Individual percent soil organic matter in each 10 cm section of each core from plots at the MRAV sites were combined into a mean value for two layers, 0–30 cm and 30–100 cm (i.e., upper vs. lower layer). This approach was based on an analysis of mean differences by depth (one-degree-of-freedom contrast: t– 3.97, p < 0.0001) [40]. Covariance estimates show that percent soil organic matter from samples taken from the upper and lower layers were not correlated (p = 0.6630) [48], so that the organic matter study was not pseudo-replicated.

#### Data preparation and statistical analysis

Mean rates of log cloth decomposition, litter decomposition and percent soil organic matter were calculated over the five plots at each site. Stepwise models were run to test differences in decomposition rates of cloth (0–10 vs. 10–30 cm; log transformed data), decomposition rates of litter (*T*. *distichum* leaf vs. wood), and differences in percent soil organic matter (0–30 vs. 30–100 cm; arcsine square root transformed data). Data were transformed to meet assumptions of normality and homogeneity on the final models when appropriate. Principal Components Analysis [48] was used to identify highly related climatic and climatic normal variables to construct principal components, which were added with the other independent variables for simultaneous testing in GLM Select models of response variable behavior across these identified principal component gradients (i.e., PrinCompS, PrinCompP, PrinComp1: cloth decomposition, litter decomposition, and soil organic matter studies, respectively; S1 Table). All possible relationships (linear and basic nonlinear effects) of covariables were entered into the Stepwise General Linear Model Selection process of PROC GLM to test all covariables simultaneously, keeping the best multiple regression model resulting in both linear and non-linear components [48]. The process uses the stepwise method to select the best model, which simultaneously adjusts for these possible effects [48]. GLM Select avoids over-parameterization by eliminating extraneous variables using stepwise procedures, and the SBC criterion adjusts for over-parameterization [51]. The final model was checked for multicollinearity by running a variance inflation factor (VIF) test [52]. The final model residuals were tested for normality and homogeneity [52].

Litter and cloth decomposition were tested against the main effects of tissue type (leaf vs. wood) or cloth depth (0–10 vs. 10–30 cm), respectively, as well as swamp type, year of study with climatic variables including: daily, annual and normal (1981–2019) [31, 41] climatic variables including: PrinCompP, PrinCompS, mean daily precipitation (mm), mean daily maximum air temperature (^o^C) [daily maximum temperature], mean daily minimum air temperature [daily minimum temperature], mean annual air temperature [annual air temperature], normal total annual precipitation [total precipitation normal] (mm), normal mean annual air temperature [air temperature normal] [31, 41], and geographical/environmental covariates of latitude, longitude, total annual precipitation [total annual precipitation], mean daily precipitation, mean daily maximum air temperature [daily maximum temperature], mean daily minimum air temperature [daily minimum temperature], pore water salinity [salinity], and annual percent time of drawdown [drawdown %] (S1 Table). The litter decomposition model had unequal variances related to class heterogeneity (site types: inland, coastal tidal and coastal nontidal), so the final model was adjusted for unequal variances using a mixed linear model procedure [48]. Note that the GLMSELECT is an approach used to determine the best subset of variables for a GLM model. In this paper, a regression (Type III Sum of Squares analysis) is presented.

Percent soil organic matter in the upper vs. lower soil layers (0–30 vs. 30–100 cm, respectively) was analyzed using the main effect of layer with each of the potential covariates (see above list for litter study) but also the climatic normal principal component (PrinComp1; S1 Table). To explore local influences in percent soil organic matter, standard errors of the percent soil organic matter were compared across latitudes by soil layers. Multiple comparisons between means of interest were constructed using a Tukey’s test. Response variables were transformed in all cases to meet assumption of ANOVA (i.e., mean CTSL, litter, and percent soil organic matter); these transformations are not subsequently mentioned. For each significant relationship between litter decomposition, cloth decomposition or percent soil organic matter responses to geographic, climatic or environmental independent variables detected by the GLMSELECT model, a summary statistic was generated and a relationship graphed using regression analysis [40].

## Results

### Decomposition of leaf and wood tissue

Decomposition of *T*. *distichum* leaf tissue during the first year was 10.9 to 6.2 X faster than wood in all swamp types respectively (leaf vs. wood: k value = 0.3468 ± 0.0154 vs. 0.0840 ± 0.0085, respectively; see also Table 1). During the second year in MRAV swamps, rates of decomposition were fairly similar (leaf vs. wood: k value = 0.3164 ± 0.0198 vs. 0.0677 ± 0.0183, respectively). Note that it was not possible to examine this pattern in the coastal sites, because by the end of the second year, there were too few litterbags remaining due to storm and animal damage (Table 1).

**Table 1:** Overall means ± SE for the decomposition rates of leaf and wood material after one and two years, half-life (50% turnover time), cloth decomposition rates, and soil organic matter %, and soil organic carbon % in MRAV inland, GOM tidal and GOM non-tidal freshwater *Taxodium distichum* swamps. Inland swamps were located in the Mississippi River Alluvial Valley from Illinois to Louisiana, and coastal tidal and nontidal from Texas to Florida in the southeastern United States. “N/A” designates that the information is not available. ‘*’ is based on one sample bag only.

			Wetland type			
Variable	MRAV inland k value	GOM nontidal k value	Coastal tidal k value	Inland half-life in yrs	Coastal nontidal half-life in yrs	Coastal tidal half-life in yrs
Litter decomposition ± SE over 1 year						
Leaves	0.3262 ± 0.0180	0.4154 ± 0.0613	0.3677 ± 0.0231	4.04 ± 1.08	2.64 ± 0.33	2.92 ± 0.49
Wood	0.0689 ± 0.0110	0.0882 ± 0.0134	0.1079 ± 0.0188	43.92 ± 5.25	17.97 ± 3.19	24.37 ± 8.18
Decomposition k value over 2 years						
Leaves	0.3157 ± 0.0202	N/A	0.3574 ± <0.0001*	3.07 ± 0.55	N/A	1.94 ± < 0.01*
Wood	0.0548 ± 0.0132	N/A	0.6973 ± <0.0001*	269.74 ± 201.12	N/A	0.99 ± < 0.01*
Cloth decomposition (log CTSL d^-1^)	3.94±0.14	2.69±0.19	3.35±0.17			
Soil organic matter % (upper layer; 0–30 cm)	5.7 ± 0.2	N/A	N/A			
Soil organic matter %(lower layer; 30–100 cm)	4.2 ± 0.2	N/A	N/A			
Soil organic carbon % (upper layer; 0–30 cm)	2.9 ± 0.1	N/A	N/A			
Soil organic carbon % (lower layer; 30–100 cm)	2.0 ± 0.1	N/A	N/A			

**Fig 3:**
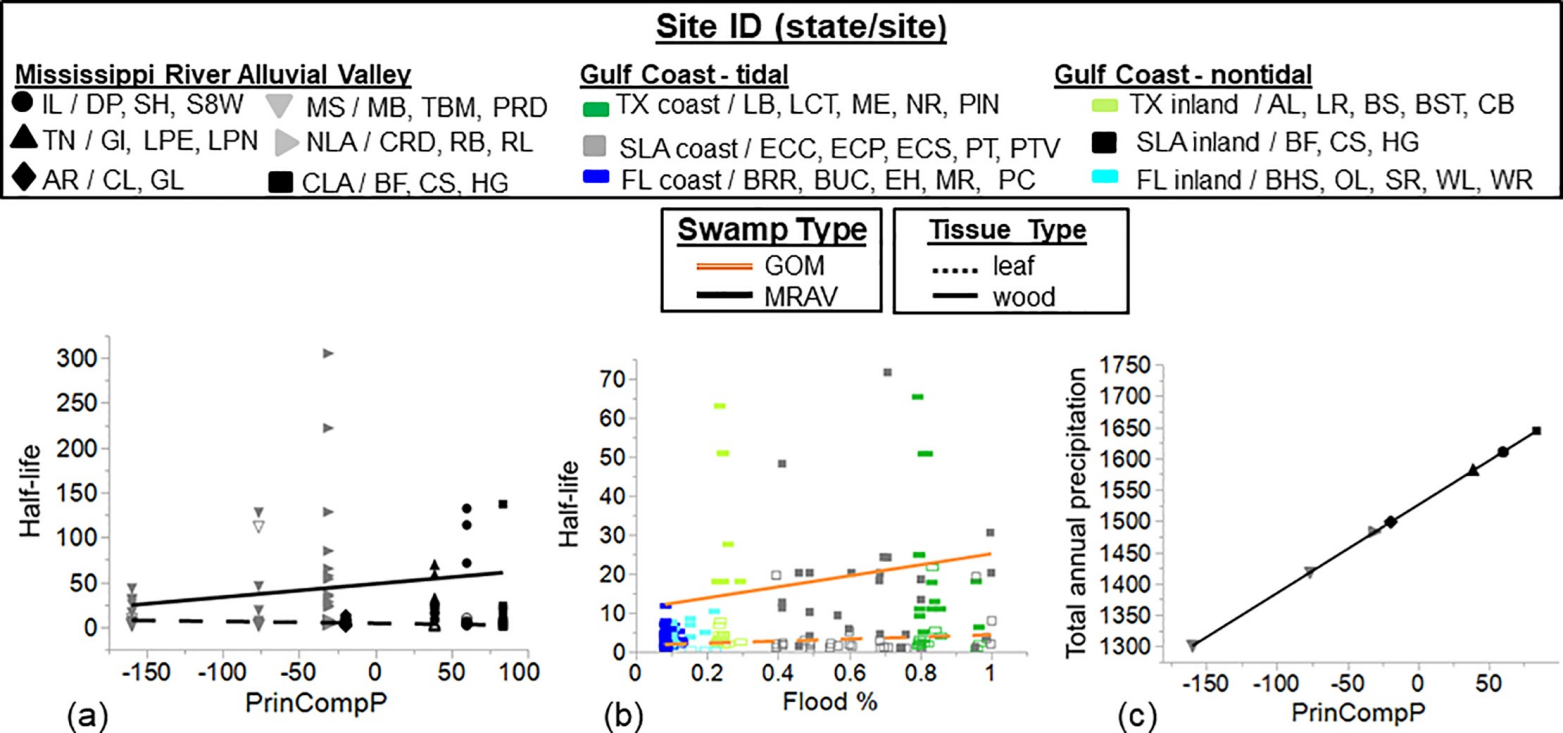
**Relationship of *T*. *distichum* leaf and wood (a-f; dashed vs. solid line, and outlined vs. solid rectangle, respectively) half-life (50% turnover time)** with best fit regressions of selected significant covariables fitting linear, log and polynomial fits of regressions for responses in the MRAV for (a) principal component of precipitation (PrinCompP; no interaction; S4 Table), and in GOM for (b) flood % (1—drawdown %; no interaction; S4 Table. Also shown is a (c) linear regression of PrinCompP with total annual precipitation. The equation for the principle component of precipitation in 2007 is PrinCompP = Total annual precipitation * 0.707107 + Mean annual precipitation * 0.707107. Regression models were prepared only for selected climatic and environmental variables identified as significant by PROC GLMSELECT (VIF < 10; S4 Table).

In the MRAV, litter decomposition rate did not have a positive relationship with the principal component of precipitation (PrinCompP), mean daily precipitation, and/or total annual precipitation (Fig 3A and S4 Table), and instead, precipitation was related to slower litter decomposition (i.e., smaller k value) as indicated by longer half-lives of the wood and leaf litter (first year data shown only). Note that the total annual precipitation and PrinComp1 were related positively (Fig 3C; r^2^ = 0.999, p < 0.0001) as well as mean daily precipitation (not shown). In GOM, as flood % increased (i.e., sites with less drawdown %), decomposition was also slower as indicated by longer half-lives of leaf and wood litter (Fig 3B and S4 Table). The pattern of decomposition for leaf vs. wood tissue was similar in all environments i.e. no interaction of tissue type with these covariables (S4 Table). Salinity levels were low MRAV inland, and GOM nontidal and tidal swamps (0.2 ± <0.1 vs. <0.1 ± <0.1 and 1.6 ± 0.1 ppt, respectively), with the exception of tidal swamps in Texas with higher salinity levels (2.9 to 5.1 ppt) than other GOM swamps. In MRAV swamps, litter decomposition was faster with increasing salinity levels of up to < 0.6 ppt (S4 Table; p = 0.0113), while in GOM swamps, litter decomposition did not differ across the range of salinity levels (p > 0.05; S4 Table).

### Cloth decomposition

Decomposition rates did not differ in cloth positioned in the upper vs. lower layers (0–10 vs. 10–30 cm, respectively) in MRAV swamps (September 2007; p > 0.05) but did differ between these layers in GOM (April 2011; p < 0.0001; S5 Table). Tidal and non-tidal site types did not differ in GOM (p > 0.05). There was no interaction of main effects in GOM (i.e., site type x layer; p > 0.05; S5 Table).

**Fig 4:**
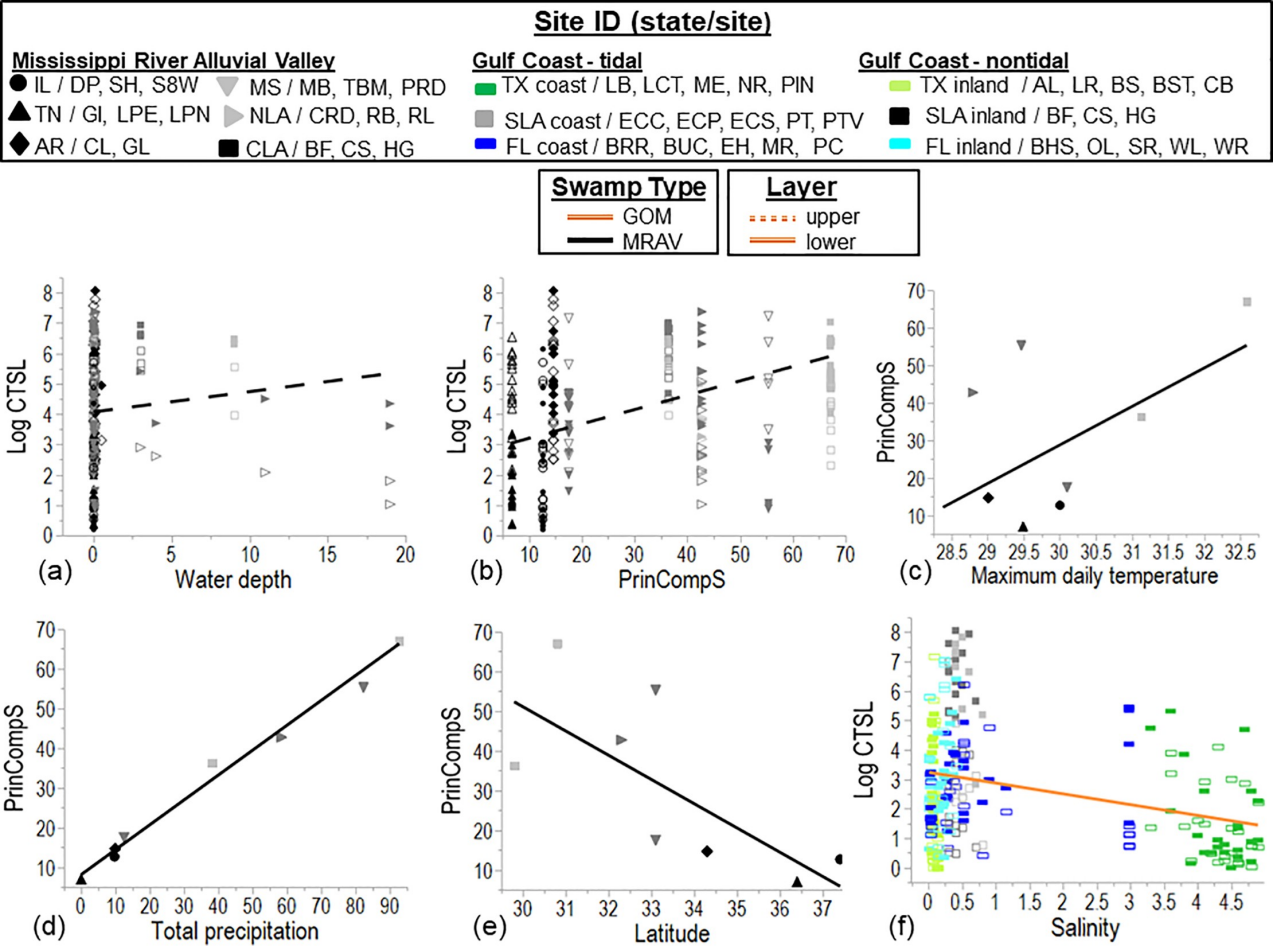
**Relationship of cloth decomposition rate (a-e), determined by cotton tensile strength loss (log CTSL day**^**-1**^**; 0–10 cm vs. 10–30 cm: upper vs. lower, respectively) with best fit regressions of significant covariables for the Mississippi River Alluvial Valley** (MRAV, 2007) including for the upper layer (lower was not significant): (a) PrinCompS, and (b) water depth (cm). The relationship of PrinCompS to (c) maximum daily temperature (^o^C), (d) total precipitation during the study (mm), and (e) latitude are given. For the Gulf of Mexico (GOM, 2011), the best fit regressions of significant covariables for both layers were combined (p > 0.05) for (e) salinity (ppt). Whole model fit for MRAV was r^2^ = 0.310, F = 16.9, p < 0.0001, and for GOM was r^2^ = 0.330, F = 28.3, p < 0.0001. PrinCompS = (mean maximum temperature * 0.567816) + (mean minimum temperature * 0.610242) + (total precipitation * 0.552439)–(mean latitude * 0.511734). Regressions models were prepared only for those climatic and environmental variables identified as significant by PROC GLMSELECT (p < 0.05; S5 Table).

Cloth decomposition decomposed more quickly in the upper layer (only) in deeper water (Fig 4A; S5 Table) and with increasing PrinCompS (Fig 4B; S5 Table). Note that PrinCompS was positively related to maximum daily temperature and total precipitation (Fig 4C and 4D, respectively; r^2^ = 0.378 and 0.988, respectively) and negatively related to latitude (Fig 4E; r^2^ = 0.589). In GOM, cloth decomposition was slower in swamps with higher salinity (Fig 4F and S5 Table) but was not related to the other environmental variables examined (S5 Table).

### Percent soil organic matter

**Fig 5:**
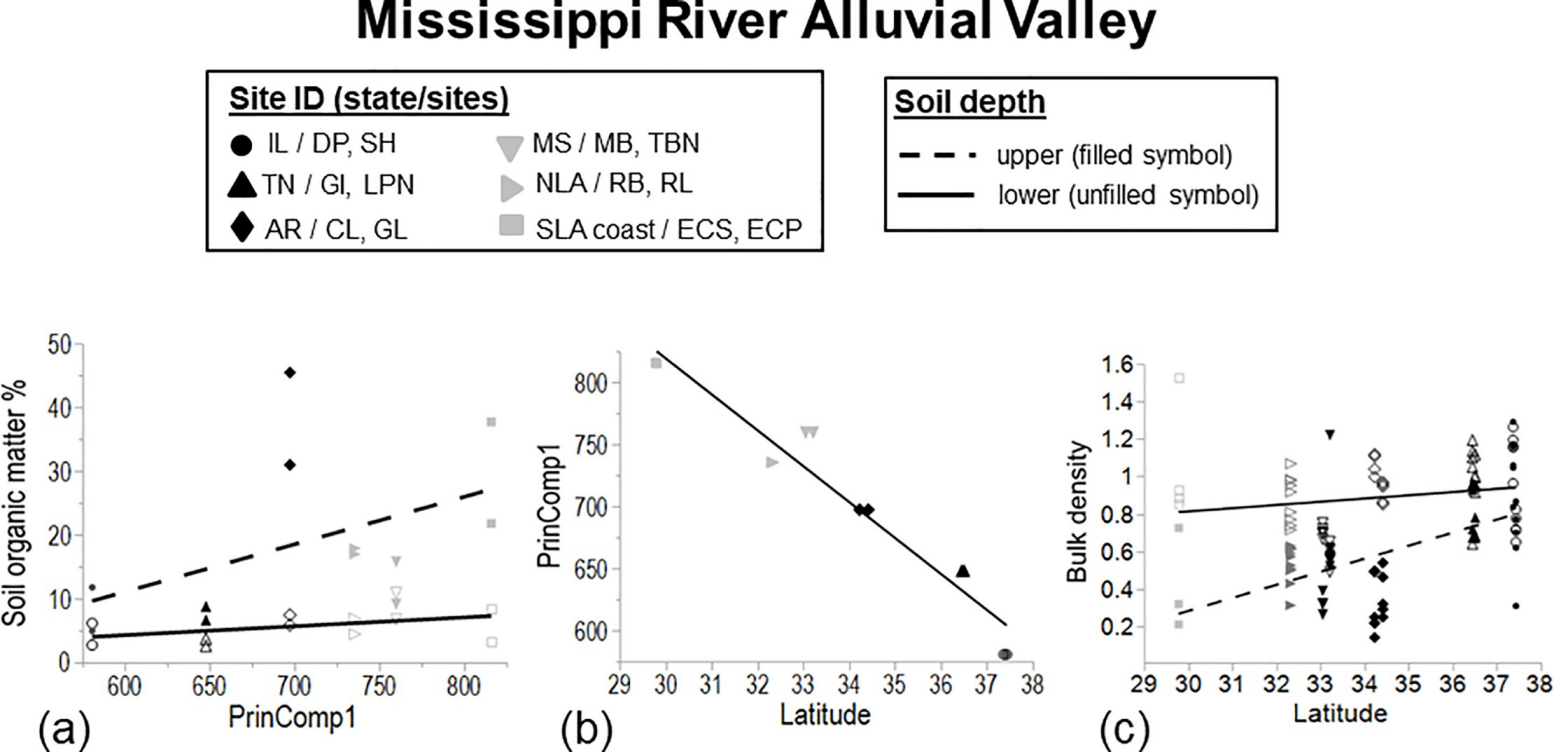
**Relationship of (a) upper and lower soil depths (0–30 vs. 30–100 cm; dashed and solid line, respectively; S6 Table) to soil organic matter % vs. PrinComp1, (b) PrinComp1 to latitude (r**^**2**^ **= 0.937, p < 0.0001), and (c) upper and lower soil depths to bulk density by latitude (r**^**2**^ **= 0.303, p < 0.0001) (S5 Table).** PrinComp1 is based on the equation: principal component 1 = (normal annual precipitation *0. 0.494947) + (normal maximum temperature *0.502801) + (normal minimum temperature *0.497784) + (normal mean temperature*0.504410). Whole model fit (MRAV only) for the organic matter % (arcsine square root transformed) was r^2^ = 0.791, F = 30.1, p < 0.0001. Regressions models were prepared only for those climatic and environmental variables identified as significant by PROC GLMSELECT (VIF < 10; S6 Table). Note that Cat Island NWR was not sampled in this survey.

In soil cored in the MRAV in 2007, percent soil organic matter was higher in the upper (0–30 cm) than the lower (30–100 cm) soil layer (p < 0.0001; S5 Table). While the main effect of Principal Component 1 (PrinComp1) was not significantly related to percent soil organic matter, this component did vary between the upper (0–30 cm) vs. lower (30–100 cm) soil layer (F = 8.5; p < 0.0001; S6 Table and Fig 5A) i.e. there was an interaction of PrinComp1 * depth. PrinComp1increased with latitude in the MRAV (r^2^ = 0.937, p < 0.0001; Fig 5B). PrinComp1was positively related to decreases in climate normal temperature and precipitation, i.e., total annual precipitation and mean, minimum and maximum air temperatures. In the MRAV, climate normal total annual precipitation and mean annual air temperature decrease with latitude, although temperatures were more moderate near the Louisiana coast than mid-latitude along the MRAV (S1 Fig). There was local variation in the percent soil organic matter in that the standard error of the mean varied among sites within a location in the upper vs. lower soil layer (r^2^ = 0.473, F = 6.0, p = 0.0044).

Percent soil organic matter did not vary across the freshwater salinities measured in the MRAV (< 0.01 and 1.1 ppt; S6 Table). None of the other normal climate or site/plot variables were related to percent soil organic matter (p > 0.05). Bulk density was lower in the upper vs. lower layer of the soil (F = 37.2, p < 0.0001), and only the upper layer increased in bulk density with latitude (r^2^ = 28.4, F = 21.8, p < 0.0001; Fig 5C).

An examination of locations with higher percent soil organic matter revealed that a few plots in mid-latitude swamps (e.g., White River NWR, Arkansas) had higher percent soil organic matter (maximum = 60.8%) than elsewhere.

### Geographical gradients of environment

Latitudinal and longitudinal gradients of environment were apparent within the *T*. *distichum* swamps of the southeastern United States. In MRAV swamps in 2007 during decomposition studies, total annual precipitation (mm) was curvilinear (latitude^2^) from south to north, while the 30-year normal annual precipitation (1981–2010) decreased northward (S1 Fig). In GOM swamps in 2011, total annual precipitation was curvilinear from west to east (Texas to Florida), with the lowest precipitation at mid-longitude (Louisiana), while normal annual precipitation increased along the same longitudinal gradient (S1 Fig).

Daily maximum temperature ^o^C in 2007 decreased northward for inland swamps, while the 30-year normal annual temperature was curvilinear and higher mid-latitude (Arkansas) than to the north or south (S1 Fig). In GOM coastal swamps, daily maximum temperatures in 2011 generally increased from west to east. In GOM swamps, daily maximum temperature generally increased from Texas to Florida in 2011; however, while the normal annual temperature had a similar trend, the normal temperatures were consistently lower than in 2011 with a mean difference of 1.7 ± 0.02 ^o^C (daily maximum temperature in 2011 vs. normal temperature: 27.4 vs. 26.0 ^o^C, respectively; S1 Fig).

These unimpounded study swamps were not flooded for much of the year including inland MRAV (n = 95), tidal GOM (n = 50) and nontidal GOM swamps (n = 30) (drawdown %: 59 ± 2%, 45 ± 3% and 82 ± 1%, respectively).

## Discussion

The idea that soil carbon stocks and litter decomposition rate are inversely linked to each was tested as the central hypothesis of this study, and a commonly held idea in discussion of soil carbon processes [6, 8, 10]. The hypothesis was generally supported by my study of *T*. *distichum* swamps of the MRAV (Table 2). In the MRAV, decomposition was slower in environments with higher precipitation in 2007 while soil organic matter % was higher southward in locations with higher 30-year normal of precipitation and temperature (>PrinComp1; Fig 5 and Table 2). While the expected overall relationship of soil carbon stocks and litter decomposition rate were inversely related as expected, the detailed relationship of environment to decomposition and geographic soil carbon stocks is generally different in these *T*. *distichum* swamps from other ecosystems.

**Table 2:** Hypothesized vs. actual direction of responses to increasing levels of independent climatic and environmental variables based on conditions during the leaf and wood decomposition (MRAV 2007 and GOM 2011; one year study), and cloth-strip decomposition (upper layer only; MRAV 2007 and GOM 2011; ten day study), or the percent soil organic matter studies (MRAV 2007). **Increase or decrease in response is depicted by “↓” vs. “↑”, respectively.** The hypothesized response is based on predictions stated in related literature. Mean air temperature and total precipitation are included. “Drawdown” is the mathematical complement of flooding (percent time the plot water depth > 1 cm. “Freshwater” indicates the low end of a salinity gradient. “Normal climate” refers to the direction of responses in the climate normal period spanning 1981–2010. “Yes” or “No” indicates if the hypothesis was supported by this study. “nr” indicates no relationship. For the 10-day cloth study, water depth was used a direct indicator of plot flooding/drawdown status. Soil organic matter % * 0.45 = soil organic carbon % [50]. The overall study hypothesis of the study is that environments that increase litter decomposition rates are related to lower soil organic matter %.

	Independent climatic or environmental variable				
Response variable	Temperature	Precipitation	Drawdown	Flooding	Freshwater
Leaf decomposition rate, MRAV	↑ / nr No	↑ / ↓ No	↑ / nr No	↓ / nr No	↑ / ↓ No
Leaf decomposition rate, GOM	↑ / nr No	↑ / ↓ No	↑ / ↑ Yes	↓ / ↓ Yes	↑ / nr Yes
Wood decomposition rate, MRAV	↑ / nr No	↑ / ↓ No	↑ / nr No	↓ / nr No	↑ / ↓ No
Wood decomposition rate, GOM	↑ / nr No	↑ / ↓ No	↑ / ↑ Yes	↓ / ↓ Yes	↑ / nr Yes
Cloth decomposition rate, MRAV	↑ / ↑ Yes	↑ / ↑ Yes	↑ / ↑ Yes	↓ / ↓ Yes	↑ / nr No
Cloth decomposition rate, GOM	↑ / ↑ Yes	↑ / ↑ Yes	↑ / ↑ Yes	↓ / ↓ Yes	↑ / ↑ Yes
Soil organic matter % (single year climate)	↓ / nr No	↓ / nr No	↓ / nr No	↑ / nr No	↑ / nr No
Soil organic matter % (normal climate)	↓ / ↑ No	↓ / ↑ No	not tested	not tested	not tested

### Decomposition and environments of the MRAV and GOM

Among other departures from expectations, *T*. *distichum* leaf and wood decomposition rates were slower in both the MRAV and GOM where water availability was higher (e.g., in either higher values of PrinCompP (principal component of precipitation-related variables) or flooding %, respectively; Fig 3, Table 2 and S4 Table). The response of decomposition to precipitation to litter decomposition was negative in this study, and different from others reported in the literature. For example, leaf decomposition rates in tropical wetlands in Costa Rica were faster in higher levels of rainfall (>5000 mm y^-1^) [53], whereas, in Panama, these rates had no relationship to precipitation [54].

Water availability may be less important to decomposition than the occurrence of well aerated and moist drawdown conditions; wetlands with fluctuating water regimes are reported to have higher leaf decomposition rates than either flooded or dry wetlands [9, 55–57]. This study was not an ideal test of the influence of flooding because none of the forty-three swamps in the study were permanently flooded. This study tested a wide range of site drawdown conditions across latitudinal and longitudinal ranges of *T*. *distichum* swamps and found that the most flooded GOM swamps had the lowest rate of decomposition (Fig 3B).

Contrary to expectations, leaf and wood decomposition rates did not increase with higher air temperature in either the MRAV or GOM (Fig 3 and S4 Table), and so was not in agreement with some other studies. For example, soil temperature was positively related to leaf decomposition and explained 95% of the variability along a tropical Andes/Amazonian lowland elevation gradient [58]. Also, leaf decomposition rate increased with air temperature and soil moisture across both latitudinal and altitudinal gradients [59–60].

The fact that wood decomposes more slowly than leaf tissue is an almost universal finding in decomposition comparisons. In this study, *T*. *distichum* wood had half-lives 10.9 to 6.2 times slower than leaves in the first year. One study comparing leaves vs. wood had decomposition rates of wood 10–53% slower than leaves of *Avicennia marina* in subtropical mangrove forests in Australia [61]. While wood decomposition studies are rare, leaf decomposition rates in this study were comparable to other regional studies (e.g., half-life = 1.04 to 2.33 y^-1^ vs. 2.45 to 4.09 y^-1^) [56] vs. this study, respectively.

Despite its lack of attention in carbon stock studies, more emphasis should be placed on the contribution of wood to soil organic matter stocks in forests with slowly decomposing wood. In this study, woody debris of *T*. *distichum* swamps had an estimated half-life of up to 300 years with a tremendous potential to remove carbon from the atmosphere semi-permanently (Fig 3). The wood of other species decomposes more quickly. For example, *Acer rubrum* wood in bottomland forests of the Congaree National Park, South Carolina had a mean half-life of 1.09 ± 0.08 years while *T*. *distichum* wood in both coastal and inland swamps in this study had much longer half-lives (mean half-life: 22.16 ± 5.45 vs. 43.92 ± 5.24 years, respectively) [62]. Another underappreciated fact is that annual rates of wood decomposition in the *T*. *distichum* forests in this study are comparable to the decomposition rates of downed wood debris of terrestrial conifer forests of the eastern U.S. (mean annual k value = 0.028) [63]. Based on the finding of this study, a discussion is warranted of the “teal” carbon potential of inland swamps from the perspective of woody *T*. *distichum* debris and soil carbon stocks maintenance [10].

While cotton strips provide a compellingly uniform material with which to study the decomposition process [37], this study provides a definitive example of why cloth decomposition studies should be used with great caution. While cotton cloth responded to environments of air temperature, precipitation and drawdown % in ways predicted by the literature and hypotheses tested in this study, the actual responses of wood and leaf litter decomposition to these environments was quite different than cloth (Table 2). Other studies have observed this same problem with proxy materials; cellulose filter degradation was little related to the decomposition response of *Quercus alba* leaves in streams [64]. These studies bring into question the validity of using proxy materials to represent plant litter responses to environment in decomposition studies, at least without careful comparisons in specific environments.

### Geographical patterns of percent soil carbon and organic matter

The idea that soil organic stocks would be higher in environments with lower litter decomposition rates was supported by this study of *T*. *distichum* swamps in the MRAV (Table 2). Soil organic matter % in these swamps was higher in geographical locations with higher values of climate normal air temperature and precipitation (PrinComp1). Leaf and wood litter decomposition rates were lowest in geographical locations with higher amounts of precipitation (PrinCompP). Many other studies support this overall relationship of soil carbon stock to litter decomposition rates [6, 8, 10]; however, litter decomposition responses in these study swamps were mostly the opposite of the expected response as based on the hypotheses and the literature (Table 2). So, while the originally stated hypothesis was supported, litter decomposition in swamps did not follow the hypothesized responses to precipitation and other environments in both the MRAV and GOM. One message from this study is that field observations are necessary to support the wetland carbon discussions.

Overall, the swamps of the MRAV have lower amounts of soil organic carbon % than better studied coastal swamps. Hansen and Nestlerode [7] reported values of soil organic carbon % for various types of Gulf Coast wetlands including fresh marsh (11.36–20.33%), freshwater pond (3.44%), brackish marsh (13.37–20.88%), salt marsh (8.15–9.33%), freshwater shrub (10.04%), hydric pine (2.04%), bottomland hardwood (8.48%), cypress-tupelo swamp (35.58%), and mangrove (23.94%). Hansen and Nestlerode [7] found that coastal *T*. *distichum* forests had mean soil organic carbon % of 35.6% at 0–10 cm depth. In the coastal Louisiana swamps of this study, this same soil depth had comparable soil organic carbon % (mean = 18.1%, range = 9.8 to 31.5%). In a swamp in Georgia (Myer’s Branch; third order stream) percent soil organic carbon was 13.2% at 7.5–13 cm depth [65]. Soil organic carbon % was somewhat lower in the *T*. *distichum* swamps of the MRAV (mean = 9.7%, range = 1.5% to 26.9%) with the exception of White River NWR in Arkansas with the highest levels of soil organic carbon % (mean = 23.5%, range = 20.4% to 26.9%). Therefore, the swamps of White River have percent soil organic carbon % comparable to that of coastal Louisiana swamps.

Coastal and inland swamp types differ, even though in the MRAV, both swamp types are underlain by clay (S2 Table). Noteworthy is that tidal swamps in coastal Louisiana are dominated by different biogeochemical factors than inland swamps (e.g., sulfate) [6]. Another considerations for the comparisons in this study is that a conversion was used (soil organic matter % * 0.45 = soil organic carbon %)[50]. Other papers cited in this paper may have used a different conversion factor [7, 66], or the measurement may have been based on gas analyzer determinations [65].

Other geographic studies comparing soil organic carbon % and geographical gradients have linked decreasing mean annual temperature northward to increased soil organic carbon accumulation [6, 8, 67]. However, this study found a strong linkage of increased climate normal air temperature and precipitation to geographic trends of increasing soil organic carbon % (using Principal Components Analysis in a GLM Select Model). Northward along latitudinal gradients of decreasing air temperature, soil organic carbon % increases in salt marsh and freshwater wetland in North America [6, 8] Craft et al. [8] suggested that soil organic carbon increased northward only in acid freshwater peatlands and not in pH neutral wetlands. The latitudinal pattern found in the MRAV analysis in this study was actually the opposite of the pattern found in these other two studies; in this study, soil organic carbon % increased southward in geographical locations with higher precipitation and temperature (PrinComp1; Table 2 and Fig 5). Outside of North America, soil organic carbon % was highest in the middle levels of precipitation (low to high precipitation gradient: lowland dry scrubland/ grassland to wet forest in a Hawaiian mountain rainshadow)[68]. A similar pattern was found in an East African semi-arid to humid arable lands including grassland and dry *Acacia* forest [69].

While it is somewhat surprising that more studies have not found that flooding is an important driver in these regional patterns, some studies underscore the importance of precipitation in the production and growth of *T*. *distichum* swamps. For example, long-term tree chronologies show that both *T*. *distichum* and *T*. *mucronatum* (Montezuma cypress in Mexico) grow faster with higher amounts of precipitation during the growing season, and that flooding is not the driver of the pattern [70]. Temperature may also be important in the production level of these forests, which could be an important environmental regulator of the supply of woody debris [23, 28]. Levels of *T*. *distichum* wood production could be the key factor in the maintenance of soil organic carbon stock, particularly because woody tissue of this species decomposes so slowly.

## Conclusions

A better understanding of carbon decomposition and stocks is invaluable to inform policies related to climate mitigation and national greenhouse gas inventories [3–4], and this study gives insight into the potential of air temperature, precipitation and other environments to influence soil carbon stocks. Combined increases in air temperature and precipitation might result in a northward increase in soil organic matter stock. At the same time, leaf and wood litter decomposition are slowed by increased water availability such as precipitation and/or flooding in *T*. *distichum* swamps of the inland Mississippi River Alluvial Valley and tidal and non-tidal northern Gulf Coast of the United States. After carbon from the atmosphere has been incorporated into tree tissue, the wood of *T*. *distichum* is very slow to decompose, especially in inland swamps (44 to 270 years). The maintenance of high levels of production [23] and tree health through water management [35] could bolster inland “teal” carbon stocks [10] in *T*. *distichum* swamps. Other factors not measured in this study undoubtedly contribute to geographical patterns in decomposition and soil organic matter stocks e.g. soil microbes, herbivore, hydrology, stream position, mineral sediment deposition, nutrient levels, storm frequency, and tides. Nevertheless, this study shows that decomposition rates and soil organic matter stocks could shift geographically shifting environments with climate and land-use change in this major North American ecosystem.

## Supporting information

S1 Table Variable abbreviations and definitions for response, climate, environment and geographic variables used in the study. (DOCX)

S2 Table Study site details for the study of leaf and wood litter decomposition, cloth decomposition, soil organic matter, environment and climate in *Taxodium distichum* var. *distichum* swamps of the southeastern United States. Site information includes hydrological unit (unit) include GOM (northern Gulf of Mexico; coastal tidal and nontidal) and MRAV (Mississippi River Alluvial Valley; inland) and type (inland, tidal, and nontidal), name of location and code, site name and code, latitude (lat) and longitude (lon) in the North American Baldcypress Swamp Network. Time periods for leaf and wood litter decomposition and cloth decomposition studies are given (litter duration). Sites with soil cores lifted with a Russian peat corer on the first day of the leaf and wood decomposition study period are designated with a “*”. A “**” indicates that the site has a Sediment Elevation Table for hydrograph correction. Pore water salinity samples were collected on each day-of-visit at all sites. A “†” indicates that the dominant trees resemble *Taxodium distichum* var. *imbricarium* based on trunk shape; however, the forests are positioned along streams or rivers, and not in isolated ponds. NOAA weather station temperature and precipitation data describe climate at sites during various studies; if two stations are listed and joined by “/”, then the second station supplies precipitation data for the statistical models [31, 41]. Flood date designates the date of highest day-of-visit water depths > 1 cm at plots from 2002–2018. Flood conditions at sites were used to set plot elevations with a USGS gage using water depth at Sediment Elevation Tables, local recorders^§^ and plots. Abbreviations include “NWR” National Wildlife Refuge, “WMA” Wildlife Management Area, “NHP&P” National Historical Park and Preserve, “NP” National Preserve and “DNR” Department of Natural Resources. “N/A” is not applicable. Aerial distance in km to the site [39] is given in brackets. Drawdown % calculated for the growing season during the deployment of the litter decomposition study (e.g., Florida: 7/26/2011-7/25/2012). Dates are given as mm/dd/yyyy.
(DOCX)

S3 Table Soil type details of study sites for the leaf and wood litter decomposition, cloth decomposition, and soil organic matter studies. Abbreviations include “NWR” National Wildlife Refuge, “WMA” Wildlife Management Area, “NHP&P” National Historical Park and Preserve, “NP” National Preserve and “DNR” Department of Natural Resources. Deep soil cores taken at site indicated with “*”. Soil information is from documents and maps on USDA NRCS websites [32–33].
(DOCX)

S4 Table Stepwise model using a mixed procedure within General Linear Models examining half-life (50% turnover time) in years and tissue type (leaf vs. wood), swamp type (inland, coastal nontidal and tidal), and group effects (swamp type x tissue type). The study was conducted in *T*. *distichum* swamps along the Mississippi River Alluvial Valley and Gulf Coast of North America in 2007 and 2011, respectively (S2 Table). Linear, log and second order polynomial relationships were fitted to significant geographic/environmental. Whole model fit was for the MRAV: F = 29.8, p < 0.0001, r^2^ = 0.733, and for GOM: F = 61.3, p < 0.0001, r^2^ = 0.689). The equation for the principle component of precipitation in 2007 was PrinCompP = Total annual precipitation * 0.707107 + Mean annual precipitation * 0.707107. Significant differences of means are based on contrasts and indicated by letters based on Tukey’s tests (p < 0.05).
(DOCX)

S5 Table Stepwise model for cotton tensile strength loss (log mean CTSL d-1) of cotton material placed underground (upper vs. lower layer: 0–10 vs. 10–30 cm, respectively) in *T. distichum* swamps located in inland settings along the Mississippi River Alluvial Valley (MRAV; 2007) and in tidal and non-tidal settings along the Gulf Coast (GOM; 2011) (S2 Table). Linear, log and second order polynomial relationships were fitted to significant geographic/environmental covariates. Whole model fits were in the MRAV in 2007 vs GOM in 2011, respectively; F = 10.7, p < 0.0001, r^2^ = 0.389 vs. F = 28.3, p < 0.0001, r^2^ = 0.319, respectively. PrinCompS = (mean maximum temperature * 0.567816) + (mean minimum temperature * 0.610242) + (total precipitation * 0.552439)–(mean latitude * 0.511734). Significant differences in means based on contrasts are indicated by letters. Relationships of individual environments identified as significant in the overall GLMSELECT model were explored using standard regression analysis with “r^2^ standard” and “p standard” reported.
(DOCX)

S6 Table Stepwise models for percent soil organic matter (arcsine square root transformed) in the upper and lower layers of the soil column (0–30 vs. 30–100 cm, respectively). Soil was cored from *T*. *distichum* swamps in September 2007 along the Mississippi River Alluvial Valley (only) (S2 Table). Mean values of organic matter ± S.E. and bulk density ± S.E. are given for the upper and lower soil layer. To examine the relationship of environment to soil organic matter, linear, log and second order polynomial relationships were fitted to significant geographic/environmental covariates including climate normal mean, maximum and minimum temperatures (^o^C), normal annual precipitation (mm) and 2007 mean daily maximum and minimum temperatures, and mean daily precipitation using the final equation: Climate Normal PC1 = (normal annual precipitation *0. 0.494947) + (normal maximum temperature *0.502801) + (normal minimum temperature *0.497784) + (normal mean temperature*0.504410). Also examined in the model were the variables: latitude, longitude, day-of-visit water depth (cm), pore water salinity (ppt), and annual percent time of drawdown. Whole model fit was: F = 30.1, p < 0.0001, r^2^ = 0.791. Significant differences of means (p < 0.001 = ‘***’; p < 0.01 = ‘**’ and p < 0.05 = “*”, and those based on contrasts were based indicated by letters. Other variables were not significantly related to the model (p > 0.05).
(DOCX)

S1 Fig Relationship of site location by latitude for the Mississippi River Alluvial Valley (MRAV, 2007; S2 Table; black line) and longitude for the Gulf of Mexico (GOM, 2011; orange line) spanning the year of the litter decomposition study (dashed line) (e.g., MRAV: October 1, 2007 –September 31, 2008) with 30-year climate normals (solid line) [31, 41]. Standard regressions are based on simple regression analysis for annual vs. normal values in the MRAV with latitude including total annual precipitation^2^ in mm (F = 85.4 vs. 657.1, respectively, r^2^ = 0.663 vs. 0.937, respectively; p < 0.0001 and p = 0.0432, respectively) and daily maximum temperature^2^ in ^o^C (F = 1367.4 vs. 276.6, respectively, r^2^ = 0.969 vs. 0.864, respectively; p = 0.0005 and p < 0.0001, respectively); and for GOM by longitude including total annual precipitation^2^ in mm (F = 1149.4 vs. 869.7, respectively, r^2^ = 0.970 vs. 0.960, respectively; p < 0.0001) and daily maximum temperature^2^ in ^o^C (longitude^-2^; F = 9870.4 vs. 1195.3, respectively, r^2^ = 0.996 vs. 0.981, respectively; p < 0.0001).
(DOCX)
