# Supplementary material for: Trends of litter decomposition and soil organic matter stocks across forested swamp environments of the southeastern US
Source: PLoS One. 2020 Jan 3;15(1):e0226998. doi: 10.1371/journal.pone.0226998 (PMC6941900; doi:10.1371/journal.pone.0226998)
Supplement: S1 Table — (DOCX) [file pone.0226998.s001.docx]

**S1 Table.** Variable abbreviations and definitions for response, climate, environment and geographic variables used in the study.

| **Variable name (units)** | **Variable abbreviation** | **Variable type** | **Definition or equation** |
| --- | --- | --- | --- |
| K value | K value | Response | y_t_ = yoe-kt; negative exponential fit of decomposing material |
| Half-life | Half-life | Response | 0.693 / k; time for 50% breakdown of decomposition material; also known as 50% turnover time |
| Cotton tensile strength percent loss day^-1^ (log mean) | CTSL | Response | CTSL = [1 – (N/C)] x 100, where N was the tensile strength of the decomposed cloth and C was the mean of the tensile strength of the control sub-strips cloth in Newtons |
| Percent soil organic matter | Soil organic matter % | Response | The average of soil organic matter of three samples in each 10 cm layer divided by the bulk density |
| Percent soil organic carbon | Soil organic carbon % | Response | Percent soil organic matter multiplied by 0.45 [50] |
| Mean daily annual precipitation (mm) | Daily precipitation | Climate | Mean daily precipitation for year long period of study |
| Mean daily maximum air temperature (^o^C) | Daily maximum temperature | Climate | Mean daily maximum air temperature for period of study |
| Mean daily minimum air temperature (^o^C) | Daily minimum temperature | Climate | Mean daily minimum air temperature for period of study |
| Principal component of precipitation and temperature | PrinCompS | Climate | PrinCompS = (mean maximum temperature * 0.567816) + (mean minimum temperature * 0.610242) + (total precipitation * 0.552439) – (mean latitude * 0.511734).  *Cloth decomposition 2007 only* |
| Mean annual air temperature (^o^C) | Daily mean temperature | Climate | Mean of the daily mean air temperature for period of study |
| Total annual precipitation (mm) | Total annual precipitation | Climate | Total precipitation for year long period of study |
| Mean annual daily precipitation (mm) | Mean annual precipitation | Climate | Mean daily precipitation for year long period of study (e.g., 2007 or 2011) |
| Total precipitation (mm) | Total precipitation | Climate | Total precipitation during the study (e.g., 8-10 days during cloth study) |
| Principal component of precipitation | PrinCompP | Climate | PrinCompP = Total annual precipitation * 0.707107 + Mean annual precipitation * 0.707107.  *Litter decomposition 2007 only* |
| Normal total annual precipitation (mm) | Normal annual precipitation | Climate Normal | 30-year average of total annual precipitation |
| Normal mean annual air temperature (^o^C) | Normal annual temperature | Climate Normal | 30-year average of mean annual temperature |
| Normal mean maximum annual air temperature (^o^C) | Normal maximum temperature | Climate Normal | 30-year average of mean maximum annual temperature |
| Normal mean minimum annual air temperature (^o^C) | Normal minimum temperature | Climate Normal | 30-year average of mean minimum annual temperature |
| Principal component of precipitation and temperature | PrinComp1 | Climate Normal | PrinComp1= (normal annual precipitation *0. 0.494947) + (normal maximum temperature *0.502801) + (normal minimum temperature *0.497784) + (normal mean temperature*0.504410).  *Soil organic matter % study* |
| Latitude | Latitude | Geographical | Latitude of study plot location |
| Longitude | Longitude | Geographical | Longitude of study plot location |
| Pore water salinity (ppt) | Salinity | Environmental | Plot pore water salinity in parts per thousand as measured on day of site visit |
| Day-of-visit water depth (cm) | Water depth | Environmental | Plot water depth in cm as measured on day of site visit |
| Annual percent time of drawdown | Drawdown % | Environmental | Plot drawdown was the percent of days the plot was drawn down (<1.0 cm of flooding) divided by the number of days of the study |
| Annual percent time of flooding | Flood % | Environmental | Mathematical complement of drawdown: 1 – drawdown % |
