# Supplementary material for: Trends of litter decomposition and soil organic matter stocks across forested swamp environments of the southeastern US
Source: PLoS One. 2020 Jan 3;15(1):e0226998. doi: 10.1371/journal.pone.0226998 (PMC6941900; doi:10.1371/journal.pone.0226998)
Supplement: S2 Table — Site information includes hydrological unit (unit) include GOM (northern Gulf of Mexico; coastal tidal and nontidal) and MRAV (Mississippi River Alluvial Valley; inland) and type (inland, tidal, and nontidal), name of location and code, site name and code, latitude (lat) and longitude (lon) in the North American Baldcypress Swamp Network. Time periods for leaf and wood litter decomposition and cloth decomposition studies are given (litter duration). Sites with soil cores lifted with a Russian peat corer on the first day of the leaf and wood decomposition study period are designated with a “*”. A “**” indicates that the site has a Sediment Elevation Table for hydrograph correction. Pore water salinity samples were collected on each day-of-visit at all sites. A “†” indicates that the dominant trees resemble Taxodium distichum var. imbricarium based on trunk shape; however, the forests are positioned along streams or rivers, and not in isolated ponds. NOAA weather station temperature and precipitation data describe climate at sites during various studies; if two stations are listed and joined by “/”, then the second station supplies precipitation data for the statistical models [31, 41]. Flood date designates the date of highest day-of-visit water depths > 1 cm at plots from 2002–2018. Flood conditions at sites were used to set plot elevations with a USGS gage using water depth at Sediment Elevation Tables, local recorders§ and plots. Abbreviations include “NWR” National Wildlife Refuge, “WMA” Wildlife Management Area, “NHP&P” National Historical Park and Preserve, “NP” National Preserve and “DNR” Department of Natural Resources. “N/A” is not applicable. Aerial distance in km to the site [39] is given in brackets. Drawdown % calculated for the growing season during the deployment of the litter decomposition study (e.g., Florida: 7/26/2011-7/25/2012). Dates are given as mm/dd/yyyy. (DOCX) [file pone.0226998.s002.docx]

**S2 Table. Study site details for the study of leaf and wood litter decomposition, cloth decomposition, soil organic matter, environment and climate in *Taxodium distichum* var. *distichum* swamps of the southeastern United States.** Site information includes hydrological unit (unit) include GOM (northern Gulf of Mexico; coastal tidal and nontidal) and MRAV (Mississippi River Alluvial Valley; inland) and type (inland, tidal, and nontidal), name of location and code, site name and code, latitude (lat) and longitude (lon) in the North American Baldcypress Swamp Network. Time periods for leaf and wood litter decomposition and cloth decomposition studies are given (litter duration). Sites with soil cores lifted with a Russian peat corer on the first day of the leaf and wood decomposition study period are designated with a “*”. A “**” indicates that the site has a Sediment Elevation Table for hydrograph correction. Pore water salinity samples were collected on each day-of-visit at all sites. A “†” indicates that the dominant trees resemble *Taxodium distichum* var. *imbricarium* based on trunk shape; however, the forests are positioned along streams or rivers, and not in isolated ponds. NOAA weather station temperature and precipitation data describe climate at sites during various studies; if two stations are listed and joined by “/”, then the second station supplies precipitation data for the statistical models [31, 41]. Flood date designates the date of highest day-of-visit water depths > 1 cm at plots from 2002-2018. Flood conditions at sites were used to set plot elevations with a USGS gage using water depth at Sediment Elevation Tables, local recorders^§^ and plots. Abbreviations include “NWR” National Wildlife Refuge, “WMA” Wildlife Management Area, “NHP&P” National Historical Park and Preserve, “NP” National Preserve and “DNR” Department of Natural Resources. “N/A” is not applicable. Aerial distance in km to the site [39] is given in brackets. Drawdown % calculated for the growing season during the deployment of the litter decomposition study (e.g., Florida: 7/26/2011-7/25/2012). Dates are given as mm/dd/yyyy.

| **Unit** | **Type** | **Location / state** | **Location (code)** | **Site name (code)** | **Lat** | **Lon** | **Litter duration** | **Cloth duration** | **Soil core** | **USGS or state recorder** | **NOAA Weather Station** | **Flood**  **date** |
| --- | --- | --- | --- | --- | --- | --- | --- | --- | --- | --- | --- | --- |
| GOM | tidal | TX | Big Thicket NP (BTNP) | Lake Bayou (LB) | 30.150 | 94.096 | 7/18/2011 to 8/7/2013 | 4/26/2011 to 5/3/2011 |  | USGS08041780^§^ [1.6 km] | GHCND:USC00410613 [18.5 km] | 7/20/2016 |
| GOM | tidal | TX | Big Thicket NP (BTNP) | Pine Ridge (PIN) | 30.143 | 94.086 | 7/18/2011 to 8/7/2013 | 4/26/2011 to 5/3/2011 |  | USGS08041780^§^ [2.9 km] | GHCND:USC00410613 [18.8 km] | 7/20/2016 |
| GOM | tidal | TX | Big Thicket NP (BTNP) | Lower Cypress Tract (LCT) | 30.134 | 94.081 | 7/18/2011 to 8/7/2013 | 4/26/2011 to 5/3/2011 |  | USGS08041780 [3.5 km] | GHCND:USC00410613 [19.1 km] | 7/20/2016 |
| GOM | tidal | TX | Big Thicket NP (BTNP) | Middle Earth (ME) | 30.140 | 94.080 | N/A | 4/26/2011 to 5/3/2011 |  | USGS08041780 [3.8 km] | GHCND:USC00410613 [19.0 km] | 7/20/2016 |
| GOM | tidal | TX | Big Thicket NP (BTNP) | Neches River (NR) | 30.130 | 94.078 | N/A | 4/26/2011 to 5/3/2011 |  | USGS08041780 [4.2 km] | GHCND:USC00410613 [18.8 km] | 7/20/2016 |
| GOM | tidal | SLA | Jean Lafitte NHP&P (JLNHP&P) | Palmetto Trail (PT) | 29.791 | 90.122 | 7/13/2011 to 8/13/2013 | 4/18/2011 to 4/29/2011 |  | CRMS 0188-H01^§^  [1.4 km] | GHCND:USC00165926 [1.4 km] | 9/22/2008 |
| GOM | tidal | SLA | Jean Lafitte NHP&P (JLNHP&P) | Palmetto Trail Visitors  (PTV) | 29.789 | 90.121 | 9/6/2007 to 9/23/2009 &  7/13/2011 to 8/13/2013 | 9/6/2007 to 9/23/2007 & 4/18/2011 to 4/29/2011 |  | CRMS 0188-H01 [1.5 km] | GHCND:USC00165926 [1.2 km] | 9/22/2008 |
| GOM | tidal | SLA | Jean Lafitte NHP&P (JLNHP&P) | Education Center Canal  (ECC) | 29.787 | 90.115 | N/A | 4/18/2011 to 4/29/2011 |  | CRMS 0234-H01 [1.4 km] | GHCND:USC00165926 [0.7 km] | 9/22/2008 |
| GOM | tidal | SLA | Jean Lafitte NHP&P (JLNHP&P) | Education Center Spur  (ECS) | 29.786 | 90.114 | 9/6/2007 to 9/22/2009 | 9/6/2007 to 9/23/2007 & 4/18/2011 to 4/29/2011 | * | CRMS 0234-H01 [1.43 km] | GHCND:USC00165926 [0.5 km] | 9/22/2008 |
| GOM | tidal | SLA | Jean Lafitte NHP&P (JLNHP&P) | Education Center Parking  (ECP) | 29.785 | 90.113 | 9/6/2007 to 9/23/2009 &  7/13/2011 to 8/13/2013 | 9/6/2007 to 9/23/2007 & 4/18/2011 to 4/29/2011 | * | CRMS 0234-H01  [1.3 km]^§^ | GHCND:USC00165926 [0.4 km] | 9/22/2008 |
| GOM | tidal | FL | St. Marks NWR (SMNWR) | Buckhorn Creek (BUC) | 30.030 | 84.470 | 7/26/2011 to 7/25/2013 | 5/10/2011 to 5/22/2011 |  | USGS0232710^§^ [3.7 km] | GHCND:USW00093805 [48.1 km] | 7/24/2012 |
| GOM | tidal | FL | St. Marks NWR (SMNWR) | Poplar Creek (PC) | 30.047 | 84.466 | N/A | 5/10/2011 to 5/22/2011 |  | USGS0232710 [2.7 km] | GHCND:USW00093805 [46.2 km] | 7/24/2012 |
| GOM | tidal | FL | Big Bend WMA (BBWMA) | Mandalay Road (MR) | 30.129 | 83.962 | 7/26/2011 to 7/25/2013 | 5/10/2011 to 5/22/2011 |  | USGS02326000^§^ [9.2 km] | GHCND:USW00093805 [45.3 km] | 7/25/2012 |
| GOM | tidal | FL | Big Bend WMA (BBWMA) | Boat Ramp Road (BRR), | 30.129 | 83.969 | 7/26/2011 to 7/25/2013 | 5/10/2011 to 5/22/2011 |  | USGS02326000 [9.7 km] | GHCND:USW00093805 [44.9 km] | 7/25/2012 |
| GOM | tidal | FL | Econfina Hickory WMA (EH) | Econfina Hickory (EH) | 30.058 | 83.894 | N/A | 5/10/2011 to 5/22/2011 |  | USGS02326000^§^ [1.1 km] | GHCND:USW00093805 [55.6 km] | 7/25/2012 |
|  |  |  |  |  |  |  |  |  |  |  |  |  |
| GOM | non-tidal | TX | Big Thicket NP (BTNP) | Beaverslide Trail (BT) | 30.576 | 94.643 | 7/19/2011 to 8/6/2013 | 4/25/2011 to 5/3/2011 |  | USGS08041000^§^ [68.9 km] | GHCND:USC00410613 [67.5 km] | 2/14/2012 |
| GOM | non-tidal | TX | Big Thicket NP (BTNP) | Lance Rosier (LR) | 30.264 | 94.513 | 7/19/2011 to 8/6/2013 | 4/25/2011 to 5/3/2011 |  | USGS08041000^§^ [40.5 km] | GHCND:USC00410613 [33.3 km] | 2/14/2012 |
| GOM | non-tidal | TX | Big Thicket NP (BTNP) | Ard Lake (AL) | 30.497 | 94.101 | 7/19/2011 to 8/6/2013 | 4/25/2011 to 5/3/2011 |  | USGS08041000 [38.1 km] | GHCND:USC00410613 [50.8 km] | 2/14/2012 |
| GOM | non-tidal | TX | Big Thicket NP (BTNP) | Crazy Bridge (CB) | 30.497 | 94.112 | N/A | 4/25/2011 to 5/3/2011 |  | USGS08041000 [38.2 km] | GHCND:USC00410613 [50.1 km] | 2/14/2012 |
| GOM | non-tidal | TX | Big Thicket NP (BTNP) | Big Sandy (BS) | 30.575 | 94.632 | N/A | 4/25/2011 to 5/3/2011 |  | USGS08041000 [68.2 km] | GHCND:USC00410613 [67.3 km] | 2/14/2012 |
| GOM | non-tidal | FL | St. Marks NWR (SMNWR) | Otter Lake (OL) | 30.025 | 84.415 | 7/26/2011 to 7/25/2013 | 5/10/2011 to 5/22/2011 |  | USGS08041000 [8.0 km] | GHCND:USW00093805 [46.8 km] | 7/24/2012 |
| GOM | non-tidal | FL | St. Marks NWR (SMNWR) | Bunkhouse Swamp | 30.167 | 84.243 | N/A | 5/10/2011 to 5/22/2011 |  | USGS08041000 [47.2 km] | GHCND:USW00093805 [29.5 km] | 7/24/2012 |
| GOM | non-tidal | FL | Aucilla WMA (AWMA) | Spur Road (SR) | 30.186 | 83.998 | 7/26/2011 to 7/25/2013 | 5/10/2011 to 5/22/2011 |  | USGS02326500^§^ [27.5 km] | GHCND:USW00093805 [38.8 km] | 7/25/2012 |
| GOM | non-tidal | FL | Aucilla WMA (AWMA) | Western Road (WR) | 30.175 | 83.977 | 7/26/2011 to 7/25/2013 | 5/10/2011 to 5/22/2011 |  | USGS02326500 [27.3 km] | GHCND:USW00093805 [40.7 km] | 7/25/2012 |
| GOM | non-tidal | FL | Aucilla WMA (AWMA) | Welaunee Landing (WL) | 30.203 | 83.955 | N/A | 5/10/2011 to 5/22/2011 |  | USGS02326500 [23.4 km] | GHCND:USW00093805 [40.3 km] | 7/25/2012 |
|  |  |  |  |  |  |  |  |  |  |  |  |  |
| MRAV | inland | IL | Illinois DNR (ILDNR) | Deer Pond (DP) | 37.428 | 88.933 | 9/16/2007 to 9/6/2009 | 9/16/2007 to 9/26/2007 | * | IL0361200^§^ [7.7 km] | GHCND:USW00093810/  GHCND:USR0000ICBO [41.7 km] | 8/25/2009 |
| MRAV | inland | IL | Illinois DNR (ILDNR) | Snake Hole (SH) | 37.367 | 88.976 | 9/16/2007 to 9/6/2009 | 9/16/2007 to 9/26/2007 | * | IL0361200^§^ [1.5 km] | GHCND:USW00093810/  GHCND:USR0000ICBO [45.4 km] | 8/25/2009 |
| MRAV | inland | IL | Illinois DNR (ILDNR) | Section 8 Woods (S8W) | 37.310 | 89.007 | 9/16/2007 to 9/6/2009 | 9/16/2007 to 9/26/2007 |  | IL0361200 [15.5 km] | GHCND:USW00093810/  GHCND:USR0000ICBO [49.5 km] | 8/25/2009 |
| MRAV | inland | KY | Reelfoot Lake NWR (RLNWR) | Long Point North (LPN) | 36.506 | 89.336 | 9/15/2007 to 9/8/2009 | 9/15/2007 to 9/25/2007 | * | USGS07027005^§^ [17.1 km] | GHCND:USC00409219 [26.7 km] | 12/13/2009 |
| MRAV | inland | TN | Reelfoot Lake NWR (RLNWR) | Long Point East (LPE) | 36.492 | 89.316 | 9/15/2007 to 9/8/2009 | 9/15/2007 to 9/25/2007 |  | USGS07027005 [18.9 km] | GHCND:USC00409219 [24.5 km] | 12/13/2009 |
| MRAV | inland | TN | Reelfoot Lake NWR (RLNWR) | Grassy Island (GI) | 36.447 | 89.349 | 9/15/2007 to 9/8/2009 | 9/15/2007 to 9/25/2007 | * | USGS07027005 [11.4 km ] | GHCND:USC00409219 [26.2 km] | 12/13/2009 |
| MRAV | inland | AR | White River NWR (WRNWR) | Goose Lake (GL) | 34.415 | 91.116 | 9/14/2007 to 9/10/2009 | 9/14/2007 to 9/24/2007 | * | USGS07077000^§^ [5.1 km] | GHCND:USC00221707 [42.3 km] | 9/08/2008 |
| MRAV | inland | AR | White River NWR (WRNWR) | Covington Lake (CL) | 34.262 | 91.095 | 9/14/2007 to 9/10/2009 | 9/14/2007 to 9/24/2007 |  | USGS07077000^§^ [16.1 km] | GHCND:USC00221707 [50.0 km] | 9/08/2008 |
| MRAV | inland | AR | White River NWR (WRNWR) | Burnt Lake (BL) | 34.228 | 91.111 | 9/14/2007 to 9/10/2009 | 9/14/2007 to 9/24/2007 | * | USGS07077000 [19.2 km] | GHCND:USC00221707 [42.5 km] | 9/08/2008 |
| MRAV | inland | MS | Morgan Brake NWR (MBNWR) | Providence Road (PRD) | 33.236 | 90.185 | 9/13/2007 to 9/11/2009 | 9/13/2007 to 9/24/2007 |  | USGS07281600 [30.3 km] | GHCND:USC00221707 [113.6 km] | 12/08/2004 |
| MRAV | inland | MS | Morgan Brake NWR (MBNWR) | Morgan Brake (MB) | 33.214 | 90.166 | 9/13/2007 to 9/11/2009 | 9/13/2007 to 9/24/2007 | * | USGS07281600 [33.3 km] | GHCND:USC00221707 [115.0 km] | 12/08/2004 |
| MRAV | inland | MS | Hillside NWR (HNWR) | Tipton Bayou North (TBN) | 33.051 | 90.282 | 9/13/2007 to 9/11/2009 | 9/13/2007 to 9/24/2007 | * | USGS07281600 [52.0 km] | GHCND:USC00229860 [24.7 km] | 12/08/2004 |
| MRAV | inland | NLA | Tensas NWR (TNWR) | Cross Roads (CR) | 32.349 | 91.322 | 9/12/2007 to 9/28/2009 | 9/12/2007 to 9/23/2007 |  | USGS07369500 [14.1 km] | GHCND:USC00168923 [14.1 km] | 9/27/2008 |
| MRAV | inland | NLA | Tensas NWR (TNWR) | Rainey Lake (RL) | 32.326 | 91.359 | 9/12/2007 to 9/28/2009 | 9/12/2007 to 9/23/2007 | * | USGS07369500 [19.9 km] | GHCND:USC00168923 [19.9 km] | 9/27/2008 |
| MRAV | inland | NLA | Tensas NWR (TNWR) | Rainey Brake (RB) | 32.321 | 91.371 | 9/12/2007 to 9/28/2009 | 9/12/2007 to 9/23/2007 | * | USGS07369500^§^ [19.9 km] | GHCND:USC00168923 [19.9 km] | 9/27/2008 |
| MRAV | inland | CLA | Cat Island NWR (CINWR) | Heavens Grove (HG) | 30.797 | 91.464 | 9/5/2007 to 9/26/2009 & 8/24/2011 to 8/14/2013 | 9/5/2007 to 9/26/2007 |  | USGS01160 [48.0 km] | GHCND:USC00168136/  GHCND:USW00013970 [41.7 km] | 4/20/2007 |
| MRAV | inland | CLA | Cat Island NWR (CINWR) | Check Station (CS) | 30.794 | 91.453 | 9/5/2007 to 9/26/2009 & 8/24/2011 to 8/14/2013 | 9/5/2007 to 9/19/2007 |  | USGS01160 [47.2 km] | GHCND:USC00168136/  GHCND:USW00013970 [40.7 km] | 4/20/2007 |
| MRAV | inland | CLA | Cat Island NWR (CINWR) | Blackfork Trail (BF) | 30.785 | 91.449 | 9/5/2007 to 9/26/2009 & 8/24/2011 to 8/14/2013 | 9/5/2007 to 9/19/2007 |  | USGS01160^§^ [46.2 km] | GHCND:USC00168136/  GHCND:USW00013970 [39.5 km] | 4/20/2007 |
