# Supplementary material for: Trends of litter decomposition and soil organic matter stocks across forested swamp environments of the southeastern US
Source: PLoS One. 2020 Jan 3;15(1):e0226998. doi: 10.1371/journal.pone.0226998 (PMC6941900; doi:10.1371/journal.pone.0226998)
Supplement: S3 Table — Abbreviations include “NWR” National Wildlife Refuge, “WMA” Wildlife Management Area, “NHP&P” National Historical Park and Preserve, “NP” National Preserve and “DNR” Department of Natural Resources. Deep soil cores taken at site indicated with “*”. Soil information is from documents and maps on USDA NRCS websites [32–33]. (DOCX) [file pone.0226998.s003.docx]

**S3 Table**. Soil type details of study sites for the leaf and wood litter decomposition, cloth decomposition, and soil organic matter studies. Abbreviations include “NWR” National Wildlife Refuge, “WMA” Wildlife Management Area, “NHP&P” National Historical Park and Preserve, “NP” National Preserve and “DNR” Department of Natural Resources. Deep soil cores taken at site indicated with “*”. Soil information is from documents and maps on USDA NRCS websites [32 ̶ 33].

| Unit | Swamp type | Location | Location (code) | Site name (code) | Lat | Lon | Soil series | Soil type | Soil core | County/  Parish |
| --- | --- | --- | --- | --- | --- | --- | --- | --- | --- | --- |
| GOM | tidal | TX | Big Thicket NP (BTNP) | Lake Bayou (LB) | 30.150 | 94.096 | Larose | Mucky peat over clay |  | Jefferson |
| GOM | tidal | TX | Big Thicket NP (BTNP) | Pine Ridge (PIN) | 30.143 | 94.086 | Larose | Mucky peat over clay |  | Jefferson |
| GOM | tidal | TX | Big Thicket NP (BTNP) | Lower Cypress Tract (LCT) | 30.134 | 94.081 | Larose | Mucky peat over clay |  | Jefferson |
| GOM | tidal | TX | Big Thicket NP (BTNP) | Middle Earth (ME) | 30.140 | 94.080 | Larose | Mucky peat over clay |  | Jefferson |
| GOM | tidal | TX | Big Thicket NP (BTNP) | Neches River (NR) | 30.130 | 94.078 | Larose | Mucky peat over clay |  | Jefferson |
| GOM | tidal | SLA | Jean Lafitte NHP&P (JLNHP&P) | Palmetto Trail (PT) | 29.791 | 90.122 | Schriever clay | Clay |  | Jefferson |
| GOM | tidal | SLA | Jean Lafitte NHP&P (JLNHP&P) | Palmetto Trail Visitors  (PTV) | 29.789 | 90.121 | Schriever clay | Clay |  | Jefferson |
| GOM | tidal | SLA | Jean Lafitte NHP&P (JLNHP&P) | Education Center Canal  (ECC) | 29.787 | 90.115 | Schriever clay | Clay |  | Jefferson |
| GOM | tidal | SLA | Jean Lafitte NHP&P (JLNHP&P) | Education Center Spur  (ECS) | 29.786 | 90.114 | Schriever clay | Clay | * | Jefferson |
| GOM | tidal | SLA | Jean Lafitte NHP&P (JLNHP&P) | Education Center Parking  (ECP) | 29.785 | 90.113 | Schriever clay | Clay | * | Jefferson |
| GOM | tidal | FL | St. Marks NWR (SMNWR) | Buckhorn Creek (BUC) | 30.030 | 84.470 | Woodville Karst plain, Gulf Coastal Lowland | Undifferentiated sand and clay |  | Wakulla |
| GOM | tidal | FL | St. Marks NWR (SMNWR) | Poplar Creek (PC) | 30.047 | 84.466 | Woodville Karst plain, Gulf Coastal Lowland | Undifferentiated sand and clay |  | Wakulla |
| GOM | tidal | FL | Big Bend WMA (BBWMA) | Mandalay Road (MR) | 30.129 | 83.962 | River Valley Lowlands | Undifferentiated sand and clay |  | Taylor |
| GOM | tidal | FL | Big Bend WMA (BBWMA) | Boat Ramp Road (BRR) | 30.129 | 83.969 | River Valley Lowlands | Undifferentiated sand and clay |  | Taylor |
| GOM | tidal | FL | Econfina Hickory WMA (EH) | Econfina Hickory (EH) | 30.058 | 83.894 | River Valley Lowlands | Undifferentiated sand and clay |  | Taylor |
|  |  |  |  |  |  |  |  |  |  |  |
| GOM | non-tidal | TX | Big Thicket NP (BTNP) | Beaverslide Trail (BT) | 30.576 | 94.643 | Hatliff-Pluck-Nahatche | Clay and loam, poorly drained |  | Polk |
| GOM | non-tidal | TX | Big Thicket NP (BTNP) | Lance Rosier (LR) | 30.264 | 94.513 | Kirbyville-Waller-Otanya | Sand over clay |  | Hardin |
| GOM | non-tidal | TX | Big Thicket NP (BTNP) | Ard Lake (AL) | 30.497 | 94.101 | Kirbyville-Waller-Otanya | Sand over clay |  | Hardin |
| GOM | non-tidal | TX | Big Thicket NP (BTNP) | Crazy Bridge (CB) | 30.497 | 94.112 | Kirbyville-Waller-Otanya | Sand over clay |  | Hardin |
| GOM | non-tidal | TX | Big Thicket NP (BTNP) | Big Sandy (BS) | 30.575 | 94.632 | Hatliff-Pluck-Nahatche | Clay and loam, poorly drained |  | Polk |
| GOM | non-tidal | FL | St. Marks NWR (SMNWR) | Otter Lake (OL) | 30.025 | 84.415 | Woodville Karst plain, Gulf Coastal Lowland | Undifferentiated sand and clay |  | Wakulla |
| GOM | non-tidal | FL | St. Marks NWR (SMNWR) | Bunkhouse Swamp | 30.167 | 84.243 | Woodville Karst plain, Gulf Coastal Lowland | Undifferentiated sand and clay |  | Wakulla |
| GOM | non-tidal | FL | Aucilla WMA (AWMA) | Spur Road (SR) | 30.186 | 83.998 | River Valley Lowlands | Undifferentiated sand and clay |  | Taylor |
| GOM | non-tidal | FL | Aucilla WMA (AWMA) | Western Road (WR) | 30.175 | 83.977 | River Valley Lowlands | Undifferentiated sand and clay |  | Taylor |
| GOM | non-tidal | FL | Aucilla WMA (AWMA) | Welaunee Landing (WL) | 30.203 | 83.955 | River Valley Lowlands | Undifferentiated sand and clay |  | Taylor |
|  |  |  |  |  |  |  |  |  |  |  |
| MRAV | inland | IL | Illinois DNR (ILDNR) | Deer Pond (DP) | 37.428 | 88.933 | Sharon-Belknap | Silt loam | * | Johnson |
| MRAV | inland | IL | Illinois DNR (ILDNR) | Snake Hole (SH) | 37.367 | 88.976 | Sharon-Belknap | Silt loam | * | Johnson |
| MRAV | inland | IL | Illinois DNR (ILDNR) | Section 8 Woods (S8W) | 37.310 | 89.007 | Karnak-Dupo | Clay & silt loam |  | Johnson |
| MRAV | inland | TN/KY | Reelfoot Lake NWR (RLNWR) | Long Point North (LPN) | 36.506 | 89.336 | Reelfoot silt loam | Silt loam & silty clay |  | Fulton |
| MRAV | inland | TN/KY | Reelfoot Lake NWR (RLNWR) | Long Point East (LPE) | 36.492 | 89.316 | Reelfoot silt loam | Silt loam & silty clay |  | Obion |
| MRAV | inland | TN/KY | Reelfoot Lake NWR (RLNWR) | Grassy Island (GI) | 36.447 | 89.349 | Reelfoot silt loam | Silt loam & silty clay | * | Obion |
| MRAV | inland | AR | White River NWR (WRNWR) | Goose Lake (GL) | 34.415 | 91.116 | Sharkey-Acadia | Clay and loam, bottomland | * | Arkansas |
| MRAV | inland | AR | White River NWR (WRNWR) | Covington Lake (CL) | 34.262 | 91.095 | Sharkey-Acadia | Clay and loam, bottomland |  | Arkansas |
| MRAV | inland | AR | White River NWR (WRNWR) | Burnt Lake (BL) | 34.228 | 91.111 | Sharkey-Acadia | Clay and loam, bottomland | * | Arkansas |
| MRAV | inland | MS | Morgan Brake NWR (MBNWR) | Providence Road (PRD) | 33.236 | 90.185 | Sharkey | Clay |  | Holmes |
| MRAV | inland | MS | Morgan Brake NWR (MBNWR) | Morgan Brake (MB) | 33.214 | 90.166 | Sharkey | Clay | * | Holmes |
| MRAV | inland | MS | Hillside NWR (HNWR) | Tipton Bayou North (TBN) | 33.051 | 90.282 | Sharkey | Clay | * | Holmes/Yazoo |
| MRAV | inland | NLA | Tensas NWR (TNWR) | Cross Roads (CR) | 32.349 | 91.322 | Tensas-Sharkey | Clay |  | Tensas |
| MRAV | inland | NLA | Tensas NWR (TNWR) | Rainey Lake (RL) | 32.326 | 91.359 | Tensas-Sharkey | Clay | * | Tensas |
| MRAV | inland | NLA | Tensas NWR (TNWR) | Rainey Brake (RB) | 32.321 | 91.371 | Tensas-Sharkey | Clay | * | Tensas |
| MRAV | inland | CLA | Cat Island NWR (CINWR) | Heavens Grove (HG) | 30.797 | 91.464 | Sharkey-Tensas-Dundee | Clay |  | West Feliciana |
| MRAV | inland | CLA | Cat Island NWR (CINWR) | Check Station (CS) | 30.794 | 91.453 | Sharkey-Tensas-Dundee | Clay |  | West Feliciana |
| MRAV | inland | CLA | Cat Island NWR (CINWR) | Blackfork Trail (BF) | 30.785 | 91.449 | Sharkey-Tensas-Dundee | Clay |  | West Feliciana |
