# Supplementary material for: Trends of litter decomposition and soil organic matter stocks across forested swamp environments of the southeastern US
Source: PLoS One. 2020 Jan 3;15(1):e0226998. doi: 10.1371/journal.pone.0226998 (PMC6941900; doi:10.1371/journal.pone.0226998)
Supplement: S4 Table — The study was conducted in T. distichum swamps along the Mississippi River Alluvial Valley and Gulf Coast of North America in 2007 and 2011, respectively (S2 Table). Linear, log and second order polynomial relationships were fitted to significant geographic/environmental. Whole model fit was for the MRAV: F = 29.8, p < 0.0001, r2 = 0.733, and for GOM: F = 61.3, p < 0.0001, r2 = 0.689). The equation for the principle component of precipitation in 2007 was PrinCompP = Total annual precipitation * 0.707107 + Mean annual precipitation * 0.707107. Significant differences of means are based on contrasts and indicated by letters based on Tukey’s tests (p < 0.05). (DOCX) [file pone.0226998.s004.docx]

**S4 Table**. Stepwise model using a mixed procedure within General Linear Models examining half-life (50% turnover time) in years and tissue type (leaf vs. wood), swamp type (inland, coastal nontidal and tidal), and group effects (swamp type x tissue type). The study was conducted in *T. distichum* swamps along the Mississippi River Alluvial Valley and Gulf Coast of North America in 2007 and 2011, respectively (S2 Table). Linear, log and second order polynomial relationships were fitted to significant geographic/environmental. Whole model fit was for the MRAV: F = 29.8, p < 0.0001, r^2^ = 0.733, and for GOM: F = 61.3, p < 0.0001, r^2^ = 0.689). The equation for the principle component of precipitation in 2007 was PrinCompP = Total annual precipitation * 0.707107 + Mean annual precipitation * 0.707107. Significant differences of means are based on contrasts and indicated by letters based on Tukey’s tests (p < 0.05).

| **Variable** | **df** | **F** | **p** | **Significance** | **Mean half-life**  **± S.E.** |
| --- | --- | --- | --- | --- | --- |
| **MRAV (2007)** | 14 | 29.8 | **<0.0001** | ******* |  |
| Tissue type | 1 | 285.3 | **<0.0001** | ******* |  |
| leaf |  |  |  |  | 4.06 ± 1.07^a^ |
| wood |  |  |  |  | 44.25 ± 6.96^b^ |
| Location | 5 | 14.4 | **<0.0001** | ******* |  |
| Tissue type * location | 5 | 5.6 | **<0.0001** | ******* |  |
| PrinCompP | 1 | 5.3 | **0.0223** | ***** |  |
| Salinity | 1 | 6.6 | **0.0113** | ***** |  |
| Longitude | ~~1~~ | 6.3 | **0.0005** | ******* |  |
|  |  |  |  |  |  |
| **GOM (2011)** | 6 | 61.3 | **<0.0001** | ******* |  |
| Tissue type | 1 | 256.1 | **<0.0001** | ******* |  |
| Leaf |  |  |  |  | 2.788 ± 0.357^a^ |
| Wood |  |  |  |  | 17.208 ± 1.711^b^ |
| Flooding % | 1 | 6.2 | **0.0138** | ***** |  |
| Location | 2 | 36.3 | **<0.0001** | ******* |  |
| Location * tissue type | 2 | 10.8 | **<0.0001** | ******* |  |
