# Supplementary material for: Trends of litter decomposition and soil organic matter stocks across forested swamp environments of the southeastern US
Source: PLoS One. 2020 Jan 3;15(1):e0226998. doi: 10.1371/journal.pone.0226998 (PMC6941900; doi:10.1371/journal.pone.0226998)
Supplement: S5 Table — Linear, log and second order polynomial relationships were fitted to significant geographic/environmental covariates. Whole model fits were in the MRAV in 2007 vs GOM in 2011, respectively; F = 10.7, p < 0.0001, r2 = 0.389 vs. F = 28.3, p < 0.0001, r2 = 0.319, respectively. PrinCompS = (mean maximum temperature * 0.567816) + (mean minimum temperature * 0.610242) + (total precipitation * 0.552439)–(mean latitude * 0.511734). Significant differences in means based on contrasts are indicated by letters. Relationships of individual environments identified as significant in the overall GLMSELECT model were explored using standard regression analysis with “r2 standard” and “p standard” reported. (DOCX) [file pone.0226998.s005.docx]

**S5 Table**. Stepwise model for cotton tensile strength loss (log mean CTSL d^-1^) of cotton material placed underground (upper vs. lower layer: 0-10 vs. 10-30 cm, respectively) in *T. distichum* swamps located in inland settings along the Mississippi River Alluvial Valley (MRAV; 2007) and in tidal and non-tidal settings along the Gulf Coast (GOM; 2011) (S2 Table). Linear, log and second order polynomial relationships were fitted to significant geographic/environmental covariates. Whole model fits were in the MRAV in 2007 vs GOM in 2011, respectively; F = 10.7, p < 0.0001, r^2^ = 0.389 vs. F = 28.3, p < 0.0001, r^2^ = 0.319, respectively. PrinCompS = (mean maximum temperature * 0.567816) + (mean minimum temperature * 0.610242) + (total precipitation * 0.552439) – (mean latitude * 0.511734). Significant differences in means based on contrasts are indicated by letters. Relationships of individual environments identified as significant in the overall GLMSELECT model were explored using standard regression analysis with “r^2^ standard” and “p standard” reported.

| Variable | df | F | p | Significance | Mean CTSL ± S.E. |
| --- | --- | --- | --- | --- | --- |
| **MRAV (2007)** | 10 | 10.7 | **<0.0001** | ******* |  |
| Layer | 1 | 2.4 | **0.1217** |  |  |
| Location | 5 | 12.0 | **<0.0001** | ******* |  |
| Water depth | 1 | 3.5 | **0.0628** |  |  |
| Water depth * layer | 1 | 5.7 | **0.0176** | ***** |  |
| PrinCompS | 1 | <0.1 | **0.8992** |  |  |
| PrinCompS * layer | 1 | 9.6 | **0.0023** | ****** |  |
|  |  |  |  |  |  |
| **GOM (2011)** | 4 | 28.3 | **<0.0001** | ******* |  |
| Layer | 1 | 23.8 | **<0.0001** | ******* |  |
| Upper |  |  |  |  | 3.70±0.14^b^ |
| Lower |  |  |  |  | 3.18±0.14^a^ |
| Salinity | 1 | 6.3 | **0.0130** | ***** |  |
| Location | 2 | 124.1 | **<0.0001** | ******* |  |
|  |  |  |  |  |  |
