# Supplementary material for: Trends of litter decomposition and soil organic matter stocks across forested swamp environments of the southeastern US
Source: PLoS One. 2020 Jan 3;15(1):e0226998. doi: 10.1371/journal.pone.0226998 (PMC6941900; doi:10.1371/journal.pone.0226998)
Supplement: S6 Table — Soil was cored from T. distichum swamps in September 2007 along the Mississippi River Alluvial Valley (only) (S2 Table). Mean values of organic matter ± S.E. and bulk density ± S.E. are given for the upper and lower soil layer. To examine the relationship of environment to soil organic matter, linear, log and second order polynomial relationships were fitted to significant geographic/environmental covariates including climate normal mean, maximum and minimum temperatures (oC), normal annual precipitation (mm) and 2007 mean daily maximum and minimum temperatures, and mean daily precipitation using the final equation: Climate Normal PC1 = (normal annual precipitation *0. 0.494947) + (normal maximum temperature *0.502801) + (normal minimum temperature *0.497784) + (normal mean temperature*0.504410). Also examined in the model were the variables: latitude, longitude, day-of-visit water depth (cm), pore water salinity (ppt), and annual percent time of drawdown. Whole model fit was: F = 30.1, p < 0.0001, r2 = 0.791. Significant differences of means (p < 0.001 = ‘***’; p < 0.01 = ‘**’ and p < 0.05 = “*”, and those based on contrasts were based indicated by letters. Other variables were not significantly related to the model (p > 0.05). (DOCX) [file pone.0226998.s006.docx]

**S6 Table.** Stepwise models for percent soil organic matter (arcsine square root transformed) in the upper and lower layers of the soil column (0-30 vs. 30-100 cm, respectively). Soil was cored from *T. distichum* swamps in September 2007 along the Mississippi River Alluvial Valley (only) (S2 Table). Mean values of organic matter ± S.E. and bulk density ± S.E. are given for the upper and lower soil layer. To examine the relationship of environment to soil organic matter, linear, log and second order polynomial relationships were fitted to significant geographic/environmental covariates including climate normal mean, maximum and minimum temperatures (^o^C), normal annual precipitation (mm) and 2007 mean daily maximum and minimum temperatures, and mean daily precipitation using the final equation: Climate Normal PC1= (normal annual precipitation *0. 0.494947) + (normal maximum temperature *0.502801) + (normal minimum temperature *0.497784) + (normal mean temperature*0.504410). Also examined in the model were the variables: latitude, longitude, day-of-visit water depth (cm), pore water salinity (ppt), and annual percent time of drawdown. Whole model fit was: F = 30.1, p < 0.0001, r^2^ = 0.791. Significant differences of means (p < 0.001 = ‘***’; p < 0.01 = ‘**’ and p < 0.05 = “*”, and those based on contrasts were based indicated by letters. Other variables were not significantly related to the model (p > 0.05).

| Variable | df | F | p | Significance | Mean percent soil  organic matter ± S.E. | Mean bulk density ± S.E. |
| --- | --- | --- | --- | --- | --- | --- |
| Model | 11 | 30.1 | **<0.0001** | ******* |  |  |
| Depth | 4 | 8.5 | **<0.0001** | ******* |  |  |
| Upper layer |  |  |  |  | 16.1±1.5^b^ | 0.59 ± 0.04^a^ |
| Lower layer |  |  |  |  | 5.5±0.5^a^ | 0.89 ± 0.03^b^ |
| Location | 4 | 20.6 | **<0.0001** | *** |  |  |
| Location * depth | 4 | 21.8 | **<0.0001** | *** |  |  |
| PrinComp1 | 1 | 0.9 | 0.3476 |  |  |  |
| PrinComp1 * depth | 1 | 8.4 | **0.0047** | ** |  |  |
