# Supplementary material for: Trends of litter decomposition and soil organic matter stocks across forested swamp environments of the southeastern US
Source: PLoS One. 2020 Jan 3;15(1):e0226998. doi: 10.1371/journal.pone.0226998 (PMC6941900; doi:10.1371/journal.pone.0226998)
Supplement: S1 Fig — Standard regressions are based on simple regression analysis for annual vs. normal values in the MRAV with latitude including total annual precipitation2 in mm (F = 85.4 vs. 657.1, respectively, r2 = 0.663 vs. 0.937, respectively; p < 0.0001 and p = 0.0432, respectively) and daily maximum temperature2 in oC (F = 1367.4 vs. 276.6, respectively, r2 = 0.969 vs. 0.864, respectively; p = 0.0005 and p < 0.0001, respectively); and for GOM by longitude including total annual precipitation2 in mm (F = 1149.4 vs. 869.7, respectively, r2 = 0.970 vs. 0.960, respectively; p < 0.0001) and daily maximum temperature2 in oC (longitude-2; F = 9870.4 vs. 1195.3, respectively, r2 = 0.996 vs. 0.981, respectively; p < 0.0001). (DOCX) [file pone.0226998.s007.docx]

**S1 Fi****g. Relationship of site location by latitude for the Mississippi River Alluvial Valley (MRAV, 2007; S2 Table; black line) and longitude for the Gulf of Mexico (GOM, 2011; orange line) spanning the year of the litter decomposition study (dashed line) (e.g., MRAV: October 1, 2007 – September 31, 2008) with 30-year climate normals (solid line) [31, 41].** Standard regressions are based on simple regression analysis for annual vs. normal values in the MRAV with latitude including total annual precipitation^2^ in mm (F = 85.4 vs. 657.1, respectively, r^2^ = 0.663 vs. 0.937, respectively; p < 0.0001 and p = 0.0432, respectively) and daily maximum temperature^2^ in ^o^C (F = 1367.4 vs. 276.6, respectively, r^2^ = 0.969 vs. 0.864, respectively; p = 0.0005 and p < 0.0001, respectively); and for GOM by longitude including total annual precipitation^2^ in mm (F = 1149.4 vs. 869.7, respectively, r^2^ = 0.970 vs. 0.960, respectively; p < 0.0001) and daily maximum temperature^2^ in ^o^C (longitude^-2^; F = 9870.4 vs. 1195.3, respectively, r^2^ = 0.996 vs. 0.981, respectively; p < 0.0001).
